# Immune targets to stop future SARS-CoV-2 variants

**DOI:** 10.1128/spectrum.02892-23

**Published:** 2023-11-15

**Authors:** Milena Silva Souza, Jéssica Pires Farias, Wilson Barros Luiz, Alexander Birbrair, Ricardo Durães-Carvalho, Luís Carlos de Souza Ferreira, Jaime Henrique Amorim

**Affiliations:** 1 Western Bahia Virology Institute, Center of Biological Sciences and Health, Federal University of Western Bahia, Barreiras, Bahia, Brazil; 2 Department of Biological Sciences, Laboratory of Applied Pathology and Genetics, State University of Santa Cruz, Ilhéus, Bahia, Brazil; 3 Department of Dermatology, School of Medicine and Public Health, University of Wisconsin–Madison, Madison, Wisconsin, USA; 4 Department of Radiology, Columbia University Medical Center, New York, New York, USA; 5 Department of Microbiology, Immunology and Parasitology, São Paulo School of Medicine, Federal University of São Paulo (UNIFESP), São Paulo, Brazil; 6 Department of Microbiology, Vaccine Development Laboratory, Biomedical Sciences Institute, University of São Paulo, São Paulo, Brazil; Universidade Federal do Rio de Janeiro, Rio de Janeiro, Brazil

**Keywords:** immune targets, SARS-CoV-2, variant of concern, conservation, epitope

## Abstract

The SARS-COV-2 variants of concern (VOCs) accumulated mutations that confer to the viral particle a higher infectivity as well as a capacity to escape neutralizing antibodies (NAbs) elicited by vaccines. In this study, we aimed to identify immune determinants that can be targeted to control future SARS-CoV-2 VOC. Using an immunoinformatics pipeline, we constructed a first data set consisting of 215 spike (S) protein amino acid sequences of the wild-type SARS-CoV-2, as well as of the Alpha, Beta, Delta, Gamma, and Omicron variants/subvariants (BA.1, BA.2, BA.4, BA.5, XBB, and BQ.1). A second data set was composed of epitope amino acid sequences for NAb as well as for T-cells involved in anti-viral activity, retrieved from the Immune Epitope Database (IEDB). Epitope conservation and population coverage analyses were carried out using the data sets. The localization of fully conserved epitopes in the S protein was performed using PyMOL. As main results, (i) fully conserved epitopes were identified: 28 for NAbs and 53 and 99 for class-I and class-II human leukocyte antigen, respectively; (ii) the fully conserved epitopes were shown to have high coverage in the world population (99.77% class combined); and (iii) the receptor-binding domain (RBD) of the S protein better balanced numbers of epitopes for NAb and T-cells, while the subunit two concentrated the highest numbers of T-cell epitopes. These results indicate that the RBD region holds different kinds of immune targets that could be used in an escape mutation-proof vaccine antigen to control future SARS-CoV-2 variants.

**IMPORTANCE:**

The emergence of SARS-CoV-2 had a major impact across the world. It is true that the collaboration of scientists from all over the world resulted in a rapid response against COVID-19, mainly with the development of vaccines against the disease. However, many viral genetic variants that threaten vaccines have emerged. Our study reveals highly conserved antigenic regions in the vaccines have emerged. Our study reveals highly conserved antigenic regions in the spike protein in all variants of concern (Alpha, Beta, Gamma, Delta, and Omicron) as well as in the wild-type virus. Such immune targets can be used to fight future SARS-CoV-2 variants.

## INTRODUCTION

The coronavirus disease 2019 (COVID-19) is caused by the *Severe acute respiratory syndrome coronavirus 2* (SARS-CoV-2) (1). Since its emergence in late 2019, more than 770 million people have been affected globally, resulting in more than 7 million deaths (2). Despite such a relevant global epidemiological impact, the numbers of COVID-19 cases have diminished, mainly due to the progress of vaccination (3, 4), which led the World Health Organization (WHO) to declare the end to COVID-19’s emergency phase (5).

From late 2020 to early 2023, many genetic SARS-CoV-2 genetic variants and variants of concern (VOCs) have emerged. The historical sequence of VOC appearance was Alpha, Beta, Gamma, Delta, and Omicron. They carry immune escape mutations in the spike protein (the main immune target, S) that confer the capacity of circumventing the neutralization activity of serum antibodies elicited by vaccines based on the wild-type SARS-CoV-2 (6 – 8). Although it is known that mRNA vaccines based on the wild-type SARS-CoV-2 induce immunological T-cell memory able to cross-recognize VOCs from Alpha to Omicron (9), the abrogation of several neutralizing antibody (NAb) epitopes, especially in Omicron, led to the development and use of mRNA-based bivalent COVID-19 vaccines. They include a component of the original virus strain aiming to provide broad protection against COVID-19 and a component of the newest viruses: subvariants of the Omicron VOC (BA.1, BA.4, and BA.5) (10, 11).

Although bivalent COVID-19 vaccines have been shown to be effective in preventing symptomatic infections (11), the WHO recognizes that the risk of new VOCs remains (5). As there is not a global effort to eradicate the disease, it is expected that new variants of SARS-CoV-2 will emerge. To deal with this future scenario, it is important to seek highly conserved immune determinants. In this study, we aimed to find SARS-CoV-2 immune targets that have been conserved since the beginning of the COVID-19 pandemic on the S protein and that are involved in anti-viral immune response.

## RESULTS

### NAb epitopes to stop future SARS-CoV-2 variants

In order to identify B-cell immune determinants that can be targeted by NAb to stop future SARS-CoV-2 variants, we carried out conservation analyses of all NAb epitopes deposited in IEDB along with a data set containing spike protein amino acid sequences representative of the main viral genetic variants in history: Wuhan (wild type), Alpha, Beta, Gamma, Delta, and Omicron. As shown in Table 1, from the nine epitopes present in the Wuhan N-terminus domain (NTD), eight were abrogated in the Omicron VOC. Although a slight increase in the number of conserved epitopes has been detected in the Alpha VOC (mutations can generate new epitopes), they decreased over time, according to the historical sequence of VOC appearance (Alpha, Beta, Gamma, Delta, and Omicron). Relevantly, none of the original NTD epitopes was fully conserved in the data set containing sequences representative of all viral variants (named All; see Table 1). A similar result was observed when NAb epitopes present in the receptor-binding domain (RBD) were studied: from the original 107 epitopes present in Wuhan, only 21 were fully conserved in Omicron or in All. Only epitopes located in the subunit 2 (S2) of the S protein tended to be conserved: from eight present in Wuhan, eight and seven were fully conserved in Omicron and All, respectively. Nevertheless, from the 124 epitopes originally present in the whole S protein of Wuhan, only 30 and 28 were fully conserved in Omicron and All, respectively. The heat map (Fig. S1) shows that there is a clear tendency for the number of fully conserved NAb epitopes to decrease along the historical sequence of VOC appearance.

**TABLE 1:** Numbers of fully conserved NAb epitopes in subunits and domains of the spike proteins of the main SARS-CoV-2 genetic variants

Domains/subunits	Wuhan^*d*^	Alpha	Beta	Delta	Gamma	Omicron	All^*e*^
NTD (S1)^*a*^	9	8	9	6	9	1	0
RBD (S1)^*b*^	107	153	85	70	53	21	21
S2^*c*^	8	10	8	8	7	8	7
Total	124	171	102	84	70	30	28

^*a*^ NTD, N-terminus domain, located in subunit 1 of the SARS-CoV-2 spike protein.

^*b*^ RBD, receptor-binding domain, located in S1.

^*c*^ S2, subunit 2 of the SARS-CoV-2 spike protein.

^*d*^ Wuhan: wild-type SARS-CoV-2.

^*e*^ All: set of spike protein amino acid sequences (*n* = 215) from wild-type SARS-CoV-2 (Wuhan) as well as from its variants of concern (VOCs) Alpha, Beta, Gamma, Delta, and Omicron.

**Fig 1:**
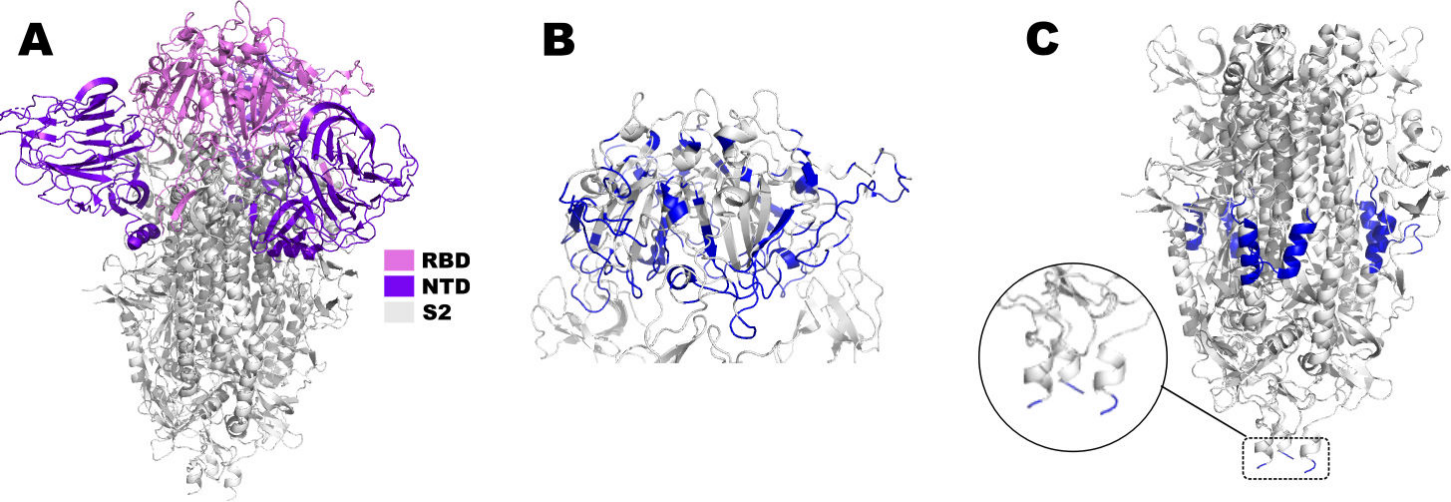
Fully conserved NAb epitopes in the spike protein of the main SARS-CoV-2 genetic variants. (A) Standard 3D model of the SARS-CoV-2 spike protein trimer. NTD, N-terminus domain (purple–blue); RBD, receptor-binding domain (violet); and S2, subunit 2 (gray). Fully conserved epitopes for neutralizing antibodies (blue) located in the RBD (**B**) and S2 (**C**).

Regarding the positions of the fully conserved NAb epitopes (considering the All data set) in the S protein, as shown using a spike protein trimer 3D model (Fig. 1A), 21 are located in the RBD (Fig. 1B). Most of these epitopes were based on amino acids in the positions 400 to 500 in the S protein amino acid sequence (Supplemental material 4). It is important to highlight that all of these epitopes are discontinuous, in contrast to the seven located in the S2 (Fig. 1C), which are all linear, and were based on amino acid positions 809 to 826 and 1,146 to 1,166 (Supplemental material 4). These regions comprise amino acid sequences before the heptad repeat 1 (HR1) and heptad repeat 2 (HR2), respectively. Four of these last-mentioned epitopes are located out of the 3D model used, which ends in the beginning of HR2 (zoomed epitopes in Fig. 1C). Collectively, these results indicate that most of the NAb epitopes present in the SARS-CoV-2 S protein were abrogated over time, according to the historical sequence of VOC appearance. In addition, most of the fully conserved NAb epitopes are located in the RBD.

### T-cell epitopes to stop future SARS-CoV-2 variants

Conservation analyses were also carried out to identify T-cell epitopes that can be used to stop future SARS-CoV-2 variants. As shown in Table 2, numbers of class-I HLA epitopes, which are targeted by IFN-γ-secreting T-cells, are given as analysis results. From the 40 epitopes present in Wuhan NTD, 19 and 12 were fully conserved in Omicron and All, respectively. From the original 30 epitopes present in the Wuhan RBD, 13 were fully conserved in Omicron and All, respectively. Epitopes contained in the S protein S1 but out of NTD or RBD decreased from eight in Wuhan to six and four in Omicron and All, respectively. In addition, epitopes originally present in the Wuhan S2 decreased from 49 to 45 and 24 in Omicron and All, respectively. Relevantly, class-I HLA epitopes decreased from 127 in Wuhan whole S protein to 83 and 53 in Omicron and All, respectively. Regarding the numbers of class-II HLA epitopes which are targeted by IFN-γ-secreting T-cells, conservation analyses revealed that (Table 3) from the 62 epitopes present in Wuhan NTD, 22 and 12 were fully conserved in Omicron and All, respectively. From the original 36 epitopes present in the Wuhan RBD, 19 were fully conserved in Omicron and All, respectively. Epitopes contained in S1 but out of NTD or RBD decreased from 27 in Wuhan to 10 and 4 in Omicron and All, respectively. In addition, epitopes originally present in the Wuhan S2 decreased from 132 to 102 and 64 in Omicron and All, respectively. Relevantly, class-II HLA epitopes decreased from 257 in Wuhan whole S protein to 150 and 99 in Omicron and All, respectively.

**TABLE 2:** Numbers of fully conserved class-I HLA epitopes in subunits and domains of the spike proteins of the main SARS-CoV-2 genetic variants

Domains/subunits	Wuhan^*e*^	Alpha	Beta	Delta	Gamma	Omicron	All^*f*^
NTD (S1)^*a*^	40	28	25	21	30	19	12
RBD (S1)^*b*^	30	39	34	31	24	13	13
S1^*c*^	8	8	9	9	09	6	4
S2^*d*^	49	39	53	38	49	45	24
Total	127	114	121	99	112	83	53^*b*^

^*a*^ NTD, N-terminus domain, located in the S1 of the SARS-CoV-2 spike protein.

^*b*^ RBD, receptor-binding domain, located in S1.

^*c*^ S1, subunit 1 of the SARS-CoV-2 spike protein.

^*d*^ S2, subunit 2 of the SARS-CoV-2 spike protein.

^*e*^ Wuhan: wild-type SARS-CoV-2.

^*f*^ All: set of spike protein amino acid sequences (*n* =215) from wild-type SARS-CoV-2 (Wuhan) as well as from its variants of concern Alpha, Beta, Gamma, Delta, and Omicron.

**TABLE 3:** Numbers of fully conserved class-II HLA epitopes in subunits and domains of the spike proteins of the main SARS-CoV-2 genetic variants

Domains/subunit	Wuhan^*e*^	Alpha	Beta	Delta	Gamma	Omicron	All^*f*^
NTD (S1)^*a*^	62	57	38	36	50	22	12
RBD (S1)^*b*^	36	63	62	49	38	16	19
S1^*c*^	27	9	23	17	20	10	4
S2^*d*^	132	131	145	115	130	102	64
Total	257	260	268	217	238	150	99

^*a*^ NTD, N-terminus domain, located in the S1 of the SARS-CoV-2 spike protein.

^*b*^ RBD, receptor-binding domain, located in S1.

^*c*^ S1, subunit 1 of the SARS-CoV-2 spike protein.

^*d*^ S2, subunit 2 of the SARS-CoV-2 spike protein.

^*e*^ Wuhan: wild-type SARS-CoV-2.

^*f*^ All: set of spike protein amino acid sequences (*n* = 215) from wild-type SARS-CoV-2 (Wuhan) as well as from its variants of concern Alpha, Beta, Gamma, Delta, and Omicron.

**Fig 2:**
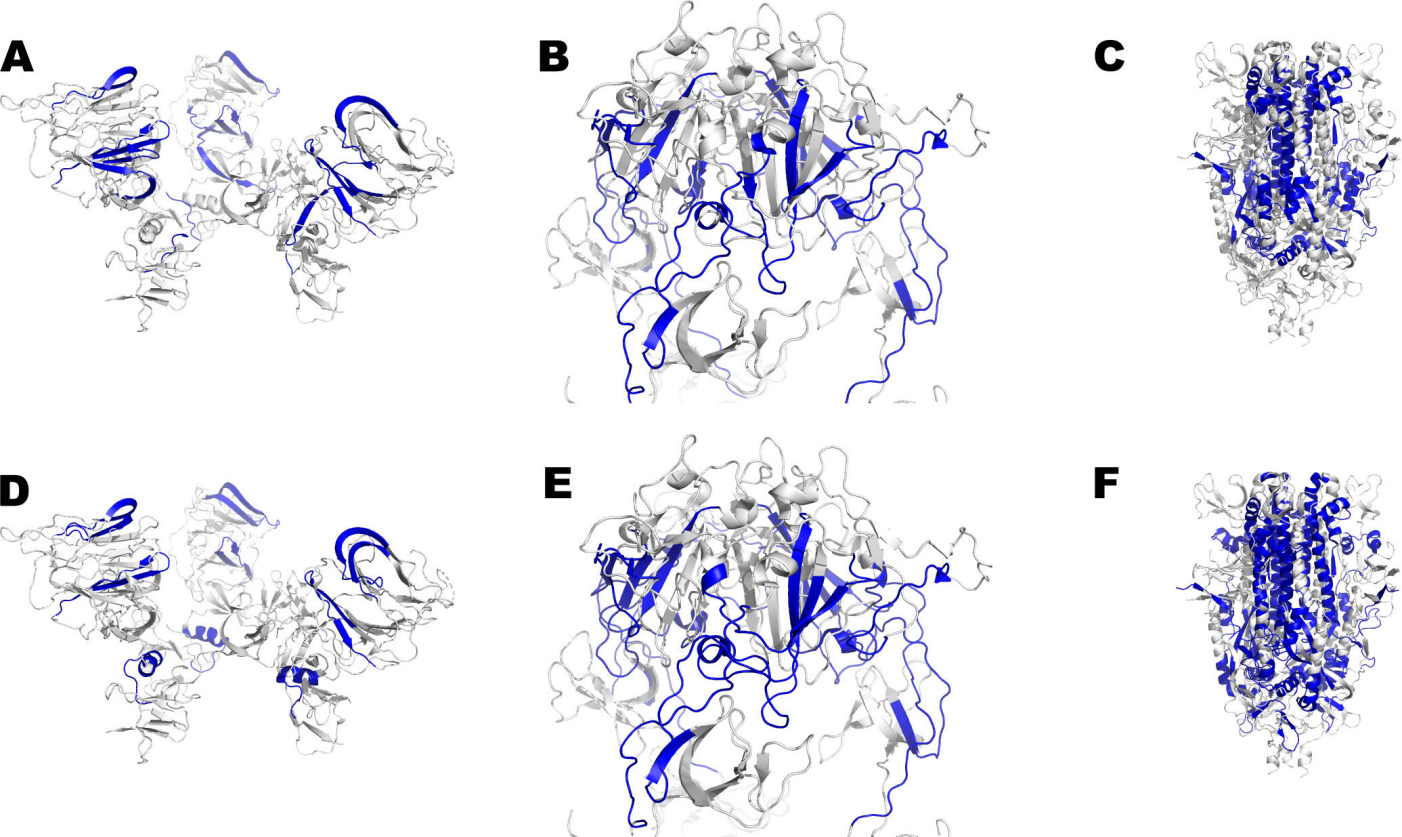
Fully conserved class-I/II HLA epitopes in the SARS-CoV-2 spike protein which are targeted by IFN-γ-secreting CD4^+^ and CD8^+^ T-cells. Epitopes are shown in blue in 3D models of the spike protein trimer domains. Fully conserved epitopes for class-I HLA, which are targeted by IFN-γ-secreting T-cells, are shown in the NTD (**A**), RBD (**B**), and S2 (**C**). Fully conserved epitopes for class-II HLA which are targeted by IFN-γ-secreting T-cells are shown in the NTD (**D**), RBD (**E**), and S2 (**F**).

It is important to highlight that all of these epitopes are linear and some of them are overlapping. The occupancy of the NTD (Fig. 2A) and RBD (Fig. 2B) by class-I HLA epitopes which are targeted by IFN-γ-secreting T-cells is lower when compared to that of S2 (Fig. 2C). In the same way, the occupancy of the NTD (Fig. 2D) and RBD (Fig. 2E) by class-II HLA epitopes which are targeted by IFN-γ-secreting T-cells is lower when compared to that of S2 (Fig. 2F). The number of class-II HLA epitopes is higher (Supplemental material 4). Although some slight increases in the number of conserved epitopes have been seen over time in the historical sequence of VOC appearance, these results collectively indicate that there was an abrogation of a relevant number of the T-cell epitopes. In addition, most of the fully conserved T-cell epitopes are located in the S2, followed by RBD and NTD. Moreover, a lower number of T-cell epitopes were abrogated when compared to NAb epitopes.

### Epitope population coverage

To estimate the coverage of the world population by T-cell fully conserved epitopes, population coverage analyses were carried out for specific populations as well as for the entire world population (Table 4). Class-I HLA epitopes which are targeted by IFN-γ-secreting T-cells were shown to have an 88.18% average coverage in the world population. The coverage was higher than 92% in most of the continents and subcontinents, except Central America. On the other hand, class-II HLA epitopes which are targeted by IFN-γ-secreting T-cells were shown to have a 59.55% average coverage in the world population. The coverage was higher than 85% in continents historically highly affected by COVID-19, such as North America and Europe. The class combined (combining class-I and class-II epitope coverages) coverage for the world population was 93.06%. Altogether, these results indicate that the fully conserved T-cell epitopes identified in this study have high coverage in the world population and have the potential to stimulate effective T-cell-based immune responses against future SARS-CoV-2 variants.

**TABLE 4:** Population coverage of class-I and class-II HLA epitopes, which are targeted by IFN-γ-secreting CD8^+^ and CD4^+^ T-cells, respectively^,^

Population/area	Class-I HLA			Class-II HLA			Class combined		
	Coverage^*a*^	Average_hit^*b*^	pc90c^*c*^	Coverage^*a*^	Average_hit^*b*^	pc90c^*c*^	Coverage^*a*^	Average_hit^*b*^	pc90c^*c*^
Central America	0.6%	0.01	0.2	31.51%	0.58	0.15	31.92%	0.59	0.15
East Asia	98.19%	8.96	2.73	55.24%	0.81	0.22	99.19%	9.76	3.69
Europe	99.64%	9.82	4.55	87.79%	1.72	0.82	99.96%	11.55	6.04
North Africa	95.11%	9.05	2.39	83.3%	1.77	0.6	99.18%	10.83	3.72
North America	98.77%	9.54	3.95	88.87%	1.68	0.9	99.86%	11.22	5.04
Northeast Asia	94.1%	5.86	2.18	49.32%	0.74	0.2	97.01%	6.6	2.4
Oceania	96.69%	6.62	2.34	46.73%	0.56	0.19	98.23%	7.18	2.64
South Africa	94.45%	8.3	2.29	30.61%	0.31	0.14	96.15%	8.61	2.43
South America	94.42%	8.8	2.25	45.79%	0.78	0.18	96.97%	9.57	2.55
South Asia	93.2%	8.06	2.19	77.16%	1.57	0.44	98.45%	9.63	3.15
Southeast Asia	96.23%	6.0	2.32	45.46%	0.64	0.18	97.94%	6.64	2.61
Southwest Asia	92.4%	7.75	2.13	55.16%	0.91	0.22	96.59%	8.65	2.45
West Indies	92.61%	5.0	2.11	77.17%	1.48	0.44	98.31%	6.47	2.81
Average^*d*^	88.19%	7.21	2.43	59.55%	1.04	0.36	93.06%	8.25	3.05
Standard deviation^*e*^	25.38%	2.54	0.97	19.92%	0.5	0.25	17.69%	2.79	1.36
World	98.77%	52.55	4.14	81.35%	1.48	0.54	99.77%	54.03	4.93

^*a*^ Population coverage (%) by Immune Epitope Database (IEDB).

^*b*^ Average number of occurrences of these epitopes in the international population by area.

^*c*^ Minimum number of epitope hits/HLA combinations recognized by 90% of the population.

^*d*^ Averages of covergare, average_hit, and pc90c values regarding all continents (population/area) studied.

^*e*^ Standard deviations of coverage, average_hit, and pc90c values regarding all continents (popullation/area) studied.

### Identification of an immune target combining epitopes for NAb and T-cells

**Fig 3:**
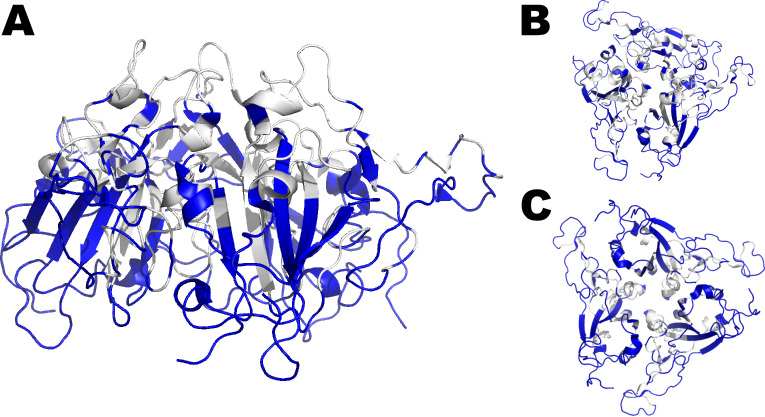
SARS-CoV-2 spike protein trimer 3D model with highlighted epitopes. (A) The fully conserved epitopes for NAb and for class-I/II HLA which are targeted by IFN-γ-secreting T-cells (shown in blue) are part of the RBD (shown in gray). (B) 3D model upper view; (C) 3D model bottom view. Most of the NAb epitopes are discontinuous. All of the T-cell epitopes are linear. Many of the epitopes are overlapping. RBD concentrates both NAb and T-cell targets that could be used in an escape mutation-proof vaccine antigen to control future SARS-CoV-2 variants.

In order to identify an immune target that could present epitopes for both NAb and T-cells, we compared the distribution of the different kinds of epitopes in NTD, RBD, and S2. All of the NAb epitopes were abrogated in the NTD, although a reduced number of T-cell epitopes have been conserved in it. In contrast, the S2 concentrated the highest number of T-cell epitopes among all domains/subunits of the S protein. However, although its NAb epitopes have been well conserved, their number was originally low when compared to RBD. This last domain concentrated most of the fully conserved NAb epitopes, whose numbers were well balanced with fully conserved T-cell epitopes when compared to the other domains. It is important to highlight that all of the NAb epitopes conserved in RBD are discontinuous. Thus, as shown in Fig. 3, the RBD was shown to better balance different kinds of immune targets that could be used in an escape mutation-proof vaccine antigen to control future SARS-CoV-2 variants.

## DISCUSSION

Although bivalent COVID-19 vaccines have been shown to be effective in preventing symptomatic infections of the newest SARS-CoV-2 genetic variants, the WHO recognizes that the risk of new VOCs remains. Many mutations were accumulated in the main vaccine target, the S protein, which turned the resulting viruses more transmissible as well as capable of circumventing the immune response elicited by vaccines. The control measures used since the beginning of the pandemic have directed the viral variant selection in these two features. As there is not a global effort to eradicate the disease, it is expected that new variants of SARS-CoV-2 will emerge from areas with absent and/or inefficient immunization programs. To deal with this future scenario, we aimed to find S protein epitopes that can be targeted to stop future variants.

Our results indicated that the highest number of fully conserved NAb epitopes is located in the RBD. In contrast, most of the T-cell conserved epitopes are located in the S2. The conservation of the NAb and T-cell epitopes in RBD and S2, respectively, may be related to key biological functions of the spike protein, especially the maintenance of structure stability, binding to the host cell receptor and membrane fusion (12). Most of the fully conserved NAb epitopes in RBD were discontinuously concentrated in positions 400 to 500 in the S protein amino acid sequence (13). These positions have key roles in the attachment to the host angiotensin-converting enzyme 2 receptor (ACE2). Mutations in these positions are expected to compromise or abrogate the attachment capacity and infection of the virus to the host cell. The S2, which is composed successively of a fusion peptide, HR1, HR2, transmembrane domain, and cytoplasmic domain fusion, is responsible for viral fusion and entry. The HR1 and HR2 are targets for proteolytic cleavages necessary to separate S1 and S2. This event is essential for fusion and entry. All of the fully conserved NAb epitopes in S2 were located before HR1 and HR2. It is expected that mutations in these positions compromise the protein stability or abrogate the fusion and entry events necessary in the viral life cycle and infectivity (14 – 16). A similar limit in mutations was previously shown by us in flaviviruses, with the most conserved epitopes for NAbs concentrated in structures with key biological functions (17, 18). The highly conserved NAb epitopes identified in this study are located in positions that seem to have also key biological functions. This result is in line with several reports showing the conservation of key epitopes in the RBD of different SARS-CoV-2 variants, and also of other coronaviruses (19 – 25). On the other hand, T-cell conserved epitopes were shown to be located in different domains of the S protein. It seems that the distribution of this last kind of epitope is not governed by the stringency related to biological functions as observed in NAb epitopes. In general, the RBD better balanced numbers of fully conserved NAb and T-cell epitopes. These results are important because both the humoral and cellular arms of the immune response are necessary to control the virus. NAbs produced by B-cells are essential in viral control because they preclude viral particles to infect cells through different mechanisms (26). On the other hand, CD8^+^ cytotoxic T-cells are capable of specifically recognizing infected cells through viral peptides presented by class-I HLA and killing them, which contributes to the control of viral synthesis and spread (27). In addition, CD4^+^ helper T-cells have the function of activating memory B-cells and CD8^+^ T-cells, contributing to a broad and more specific and effective immune response (27). As can be noted, the functions of the mentioned immune cell types are interconnected, and this highlights the importance of our finding showing that their target epitopes are conserved and more equitably present in the SARS-CoV-2 RBD.

The distribution nature of highly conserved epitopes in the S protein shown in this study can foster the rationale of future vaccine antigens. For example, the recent strategy based on the update of vaccine antigen could be replaced with a broadly protective antigen designed to deal with future SARS-CoV-2 in a long-term way. Immune targets identified here could be used to fight not only the future COVID-19 but also other coronaviruses, which are related to SARS-CoV-2, or even descend from it. In addition, combinations of fully conserved epitopes or the RBD, which better balances Nab and T-cell epitopes, could be incorporated in cutting-edge vaccine platforms, such as those based on self-amplifying RNA, which requires smaller doses and are capable of inducing potent immune responses (28). Peptides based on fully conserved epitopes of the RBD could also be studded with ferritin or other nanoparticles in order to generate more potent immune responses (28). Interestingly, ferritin self-assembles into a sphere and can be studded with proteins. All of the results and indications presented in this study are useful to fight COVID-19 in a long-term way.

One could argue that the sample size of protein sequences used in this study was not adequate. However, some important points deserve to be highlighted: (i) the uneven quality of sequences deposited into GenBank and Global Initiative on Sharing All Influenza Data (GISAID) databases and (ii) the high genomic similarity of respective SARS-CoV-2 VOCs, which can translate into a high proportion of sequences of each variant being mostly identical. The third and last point falls on the treatment given to the sequences, such as the absence of degenerate bases and duplications among VOCs. In addition, we studied only immune targets located in the SARS-CoV-2 S protein. There are other epitopes in other viral proteins. However, the amount of well-characterized epitopes in the S protein is far higher than in any other protein. Moreover, we studied only SARS-CoV-2 S proteins and not from other sarbecoviruses. Finally, one could argue that we did not present an *in vivo* proof of concept, which is true. However, all of the epitopes presented in this are real and were previously well characterized *in vitro* and *in vivo* and are involved in protective anti-viral immune mechanisms against SARS-CoV-2.

In this study, we presented robust data supported by proper analyses showing that the RBD better balances fully conserved epitopes for NAbs, as well as for IFN-γ-producing T-cells among all S protein domains. These findings indicate that the RBD concentrates the more balanced numbers of immune target kinds that have been conserved since the beginning of the COVID-19 pandemic and that could be present in an escape mutation-proof vaccine antigen.

## MATERIALS AND METHODS

### Data sets of SARS-CoV-2 spike protein amino acid sequences and epitopes

A first data set of 215 amino acid sequences of the spike protein from the wild-type SARS-CoV-2 (Wuhan, *n* = 28) as well as from its variants of concern Alpha (*n* = 24), Beta (*n* = 16), Gamma (*n* = 32), Delta (*n* = 50), and Omicron (BA.1, BA.2, BA.4, BA.5, XBB, and BQ.1, *n* = 60) was built (Supplemental material 1). Quality-filtered data sets (0% of degenerated bases and duplicate sequences) were obtained through biopython‐based software Sequence Cleaner (https://github.com/metageni/Sequence-Cleaner). Sequences were representative of the Americas, Europe, Africa, Asia, and Oceania. From July 2022 to January 2023, they were retrieved from the National Center for Biotechnology Information (NCBI) (https://www.ncbi.nlm.nih.gov/) as amino acid sequences or from the GISAID (https://gisaid.org/) as genomic nucleotide sequences. S protein-coding sequences retrieved from GISAID were translated into amino acid sequences using UGENE v.45.0 bioinformatics multiplatform (http://ugene.net/). The criteria for selecting the S protein sequences were as follows: (i) complete sequences and (ii) absence of unidentified amino acids. In addition, three other data sets of immune targets (epitope amino acid sequences) were built. Sequences were retrieved from IEDB (https://www.iedb.org/). This platform permits the retrieval of experimentally confirmed data on epitopes for antibodies and T-cell for different infectious diseases. The data set of immune targets for NAbs consisted of 415 epitopes validated by virus neutralization assays, such as plaque reduction neutralization tests, focus reduction neutralization tests, and structural biology analyses (29) (Supplemental material 2). The data set of epitopes for class-I human leukocyte antigen (HLA) consisted of 159 epitopes validated by T-cell IFN-γ release assays (Supplemental material 3). The data set of epitopes for class-II HLA consisted of 321 epitopes validated by T-cell IFN-γ release assays (Supplemental material 3). Databases (NCBI, GISAID, and IEDB) were accessed until 30 January 2023 in order to construct the data sets used in this study. Replicates of the same epitope sequences were removed from analyses. NAb epitopes with more than one chain were also removed from analyses.

### Epitope conservation analysis

As previously described (17, 18, 30), the IEDB conservation analysis tool (http://tools.iedb.org/conservancy) was used to determine epitope conservation among SARS-CoV-2 S protein sequences contained in our data set. In the present study, only fully conserved epitopes were considered (100% conserved in the S protein amino acid sequences of data sets used), including epitopes for B- and T-cells. One-to-one epitope conservation analyses using S protein data sets of Wuhan, Alpha, Beta, Gamma, Delta, or Omicron as well as using the whole data set containing all SARS-CoV-2 variant S protein amino acid sequences were carried out.

### Population coverage analysis

The T-cell epitopes selected in the conservation analysis were subjected to population coverage analysis using the IEDB population coverage calculation tool (http://tools.iedb.org/population/), as previously described (18).

### Structural biology analysis

An S protein 3D model (https://doi.org/10.2210/pdb7DDD/pdb) retrieved from the Protein Data Bank (,https://www.rcsb.org) was used to localize fully conserved epitopes for NAbs, as well as for class-I/II HLA, using PyMol (https://pymol.org/2/), as previously described (18, 30).

## Data Availability

Data sets used in this study are available as supplemental material. Accession numbers (ncbi OR gisaid) of S proteins are available in the headers of sequences in Supplemental material 1. Epitope ID for NAb and T-cell epitopes are available in Supplemental material 2 and Supplemental material 3, respectively. Results of analyses are shown in the main text, tables, and figures.

## SUPPLEMENTAL MATERIAL

Supplemental material 1 spectrum.02892-23-s0001.docx Data set of amino acid sequences of SARS-CoV-2 spike protein.

Supplemental material 2 spectrum.02892-23-s0002.docx Amino acid sequences of NAb epitopes.

Supplemental material 3 spectrum.02892-23-s0003.docx Amino acid sequences of T-cell epitopes.

Fig. S1 spectrum.02892-23-s0004.tif Heat map of NAb epitopes fully conserved in Wuhan (WT), Alpha, Beta, Gamma, Delta, and Omicron VOCs.

Supplemental legend spectrum.02892-23-s0005.docx Legend for Fig. S1.

ASM does not own the copyrights to Supplemental Material that may be linked to, or accessed through, an article. The authors have granted ASM a non-exclusive, world-wide license to publish the Supplemental Material files. Please contact the corresponding author directly for reuse.

## References

[1] Gorbalenya AE , Baker SC , Baric RS , de Groot RJ , Drosten C , Gulyaeva AA , Haagmans BL , Lauber C , Leontovich AM , Neuman BW , Penzar D , Perlman S , Poon LLM , Samborskiy DV , Sidorov IA , Sola I , Ziebuhr J , Coronaviridae Study Group of the International Committee on Taxonomy of Viruses . 2020. The species severe acute respiratory syndrome-related coronavirus: classifying 2019-nCoV and naming it SARS-Cov-2. Nat Microbiol 5:536–544. doi:10.1038/s41564-020-0695-z 32123347 PMC7095448

[2] WHO Coronavirus (COVID-19) dashboard | WHO Coronavirus disease (COVID-19) dashboard. n.d.

[3] Santos CVBD , Valiati NCM , Noronha TG de , Porto VBG , Pacheco AG , Freitas LP , Coelho FC , Gomes MF da C , Bastos LS , Cruz OG , Lana RM , Luz PM , Carvalho LMF de , Werneck GL , Struchiner CJ , Villela DAM . 2023. The effectiveness of COVID-19 vaccines against severe cases and deaths in Brazil from 2021 to 2022: a registry-based study. Lancet Reg Health Am 20:100465. doi:10.1016/j.lana.2023.100465 36936517 PMC10010656

[4] Tregoning JS , Flight KE , Higham SL , Wang Z , Pierce BF . 2021. Progress of the COVID-19 vaccine effort: viruses, vaccines and variants versus efficacy, effectiveness and escape. 10. Nat Rev Immunol 21:626–636. doi:10.1038/s41577-021-00592-1 34373623 PMC8351583

[5] Lenharo M . 2023. WHO declares end to COVID-19’s emergency phase. Nature. doi:10.1038/d41586-023-01559-z 37147368

[6] Harvey WT , Carabelli AM , Jackson B , Gupta RK , Thomson EC , Harrison EM , Ludden C , Reeve R , Rambaut A , COVID-19 Genomics UK (COG-UK) Consortium, Peacock SJ , Robertson DL . 2021. SARS-CoV-2 variants, spike mutations and immune escape. Nat Rev Microbiol 19:409–424. doi:10.1038/s41579-021-00573-0 34075212 PMC8167834

[7] Dejnirattisai W , Huo J , Zhou D , Zahradník J , Supasa P , Liu C , Duyvesteyn HME , Ginn HM , Mentzer AJ , Tuekprakhon A , et al. . 2022. SARS-CoV-2 omicron-B.1.1.529 leads to widespread escape from neutralizing antibody responses. Cell 185:467–484. 35081335 10.1016/j.cell.2021.12.046 PMC8723827

[8] Cao Y , Wang J , Jian F , Xiao T , Song W , Yisimayi A , Huang W , Li Q , Wang P , An R , et al. . 2022. Omicron escapes the majority of existing SARS-CoV-2 neutralizing antibodies. Nature 602:657–663. doi:10.1038/s41586-021-04385-3 35016194 PMC8866119

[9] Tarke A , Coelho CH , Zhang Z , Dan JM , Yu ED , Methot N , Bloom NI , Goodwin B , Phillips E , Mallal S , Sidney J , Filaci G , Weiskopf D , da Silva Antunes R , Crotty S , Grifoni A , Sette A . 2022. SARS-CoV-2 vaccination induces immunological T cell memory able to cross-recognize variants from alpha to Omicron. Cell 185:847–859. doi:10.1016/j.cell.2022.01.015 35139340 PMC8784649

[10] Commissioner O of the . 2023. COVID-19 Bivalent vaccines. FDA

[11] Link-Gelles R , Ciesla AA , Fleming-Dutra KE , Smith ZR , Britton A , Wiegand RE , Miller JD , Accorsi EK , Schrag SJ , Verani JR , Shang N , Derado G , Pilishvili T . 2022. Effectiveness of bivalent mRNA vaccines in preventing symptomatic SARS-CoV-2 infection - increasing community access to testing program, United States, september-november 2022. MMWR Morb Mortal Wkly Rep 71:1526–1530. doi:10.15585/mmwr.mm7148e1 36454688 PMC9721148

[12] Huang Y , Yang C , Xu X , Xu W , Liu S . 2020. Structural and functional properties of SARS-CoV-2 spike protein: potential antivirus drug development for COVID-19. 9. Acta Pharmacol Sin 41:1141–1149. doi:10.1038/s41401-020-0485-4 32747721 PMC7396720

[13] Yan R , Zhang Y , Li Y , Xia L , Guo Y , Zhou Q . 2020. Structural basis for the recognition of SARS-CoV-2 by full-length human ACE2. Science 367:1444–1448. doi:10.1126/science.abb2762 32132184 PMC7164635

[14] Majumdar P , Niyogi S . 2021. SARS-CoV-2 mutations: the biological trackway towards viral fitness. Epidemiol Infect 149:e110. doi:10.1017/S0950268821001060 33928885 PMC8134885

[15] Volz E . 2023. Fitness, growth and transmissibility of SARS-CoV-2 genetic variants. 10. Nat Rev Genet 24:724–734. doi:10.1038/s41576-023-00610-z 37328556

[16] Obermeyer F , Jankowiak M , Barkas N , Schaffner SF , Pyle JD , Yurkovetskiy L , Bosso M , Park DJ , Babadi M , MacInnis BL , Luban J , Sabeti PC , Lemieux JE . 2022. Analysis of 6.4 million SARS-CoV-2 genomes identifies mutations associated with fitness. Science 376:1327–1332. doi:10.1126/science.abm1208 35608456 PMC9161372

[17] Pinheiro JR , Camilo Dos Reis E , Souza R da SO , Rocha ALS , Suesdek L , Azevedo V , Tiwari S , Rocha BGS , Birbrair A , Méndez EC , Luiz WB , Amorim JH . 2021. Comparison of neutralizing dengue virus B cell epitopes and protective T cell epitopes with those in three main dengue virus vaccines. Front Immunol 12:715136. doi:10.3389/fimmu.2021.715136 34489965 PMC8417696

[18] dos Santos Franco L , Gushi LT , Luiz WB , Amorim JH . 2019. Seeking flavivirus cross-protective immunity. Front Immunol 10:2260. doi:10.3389/fimmu.2019.02260 31616432 PMC6763598

[19] Huang K-YA , Chen X , Mohapatra A , Nguyen HTV , Schimanski L , Tan TK , Rijal P , Vester SK , Hills RA , Howarth M , Keeffe JR , Cohen AA , Kakutani LM , Wu Y-M , Shahed-Al-Mahmud M , Chou Y-C , Bjorkman PJ , Townsend AR , Ma C . 2023. Structural basis for a conserved neutralization epitope on the receptor-binding domain of SARS-CoV-2. 1. Nat Commun 14:311. doi:10.1038/s41467-023-35949-8 36658148 PMC9852238

[20] Lan J , Ge J , Yu J , Shan S , Zhou H , Fan S , Zhang Q , Shi X , Wang Q , Zhang L , Wang X . 2020. Structure of the SARS-CoV-2 spike receptor-binding domain bound to the ACE2 receptor. Nature 581:215–220. doi:10.1038/s41586-020-2180-5 32225176

[21] Geanes ES , LeMaster C , Fraley ER , Khanal S , McLennan R , Grundberg E , Selvarangan R , Bradley T . 2022. Cross-reactive antibodies elicited to conserved epitopes on SARS-CoV-2 spike protein after infection and vaccination. 1. Sci Rep 12:6496. doi:10.1038/s41598-022-10230-y 35444221 PMC9019795

[22] Li T , Xue W , Zheng Q , Song S , Yang C , Xiong H , Zhang S , Hong M , Zhang Y , Yu H , et al. . 2021. Cross-neutralizing antibodies bind a SARS-CoV-2 cryptic site and resist circulating variants. 1. Nat Commun 12:5652. doi:10.1038/s41467-021-25997-3 34580306 PMC8476643

[23] Kleanthous H , Silverman JM , Makar KW , Yoon I-K , Jackson N , Vaughn DW . 2021. Scientific rationale for developing potent RBD-based vaccines targeting COVID-19. NPJ Vaccines 6:128. doi:10.1038/s41541-021-00393-6 34711846 PMC8553742

[24] Yuan M , Wu NC , Zhu X , Lee C-C , So RTY , Lv H , Mok CKP , Wilson IA . 2020. A highly conserved cryptic epitope in the receptor binding domains of SARS-CoV-2 and SARS-CoV. Science 368:630–633. doi:10.1126/science.abb7269 32245784 PMC7164391

[25] Chen Y , Zhao X , Zhou H , Zhu H , Jiang S , Wang P . 2023. Broadly neutralizing antibodies to SARS-CoV-2 and other human coronaviruses. 3. Nat Rev Immunol 23:189–199. doi:10.1038/s41577-022-00784-3 36168054 PMC9514166

[26] Burton DR . 2023. Antiviral neutralizing antibodies: from in vitro to in vivo activity. Nat Rev Immunol:1–15. doi:10.1038/s41577-023-00858-w 37069260 PMC10108814

[27] Li Q , Wang Y , Sun Q , Knopf J , Herrmann M , Lin L , Jiang J , Shao C , Li P , He X , et al. . 2022. Immune response in COVID-19: what is next? Cell Death Differ 29:1107–1122. doi:10.1038/s41418-022-01015-x 35581387 PMC9110941

[28] Callaway E . 2023. The next generation of coronavirus vaccines: a graphical guide. Nature 614:22–25. doi:10.1038/d41586-023-00220-z 36726000

[29] Peters B , Sidney J , Bourne P , Bui H-H , Buus S , Doh G , Fleri W , Kronenberg M , Kubo R , Lund O , Nemazee D , Ponomarenko JV , Sathiamurthy M , Schoenberger S , Stewart S , Surko P , Way S , Wilson S , Sette A . 2005. The immune epitope database and analysis resource: from vision to blueprint. PLOS Biol. 3:e91. doi:10.1371/journal.pbio.0030091 15760272 PMC1065705

[30] Dos Santos Franco L , Oliveira Vidal P , Amorim JH . 2017. In silico design of a zika virus non-structural protein 5 aiming vaccine protection against zika and dengue in different human populations. J Biomed Sci 24:88. doi:10.1186/s12929-017-0395-z 29169357 PMC5701345

