# Supplementary material for: Immune targets to stop future SARS-CoV-2 variants
Source: Microbiol Spectr. 2023 Nov 15;11(6):e02892-23. doi: 10.1128/spectrum.02892-23 (PMC10714790; doi:10.1128/spectrum.02892-23)
Supplement: Supplemental material 2 — Amino acid sequences of NAb epitopes. [file spectrum.02892-23-s0002.docx]

Supplementary material 2 – Amino acid sequences of NAb epitopes.

| **NAb Epitope dataset** | |
| --- | --- |
| Epitope ID | Description |
| 997006 | Y369,N370,S371,A372,F374,F377,K378,C379,Y380,G381,V382,S383,P384,T385,K386,L390,F429,T430,F515,E516,L517 |
| 1071808 | PSKPSKRSFIEDLLFNKV |
| 1074326 | A475,V483,F486,S494 |
| 1074327 | Y369,N370,A372,F374,T376,F377,K378,Y380,V382,P384,T385,K386,D389,L390,F392,D428,F429,T430,F515,L517,H519 |
| 1075135 | R346,K444,G446,G447,N448,Y449,N450,L452,V483,E484,G485,F490,S494 |
| 1075136 | R403,Q409,T415,G416,K417,D420,Y421,L455,F456,R457,K458,S459,N460,Y473,Q474,A475,G476,S477,F486,N487,Y489,F490,Q493,Y495,G496,Q498,N501,G502,Y505 |
| 1083498 | R403,D405,E406,R408,Q409,T415,G416,K417,D420,Y421,L455,F456,R457,K458,N460,Y473,Q474,A475,G476,S477,F486,N487,Y489,Q493,Y495,G502,Y505 |
| 1087140 | G446,Y449,E484,G485,F486,Y489,F490,L492,Q493,S494,G496,Q498,N501,Y505 |
| 1087186 | Y369,N370,S375,T376,F377,K378,C379,Y380,G381,V382,S383,P384,T385,K386,L390,F392,D428,T430,E516,L517 |
| 1087266 | R403,D405,R408,T415,G416,K417,D420,Y421,Y453,L455,F456,R457,K458,S459,N460,Y473,Q474,A475,G476,S477,F486,N487,Y489,Q493,S494,Y495,G496,Q498,T500,N501,G502,Y505 |
| 1087267 | R403,D405,T415,G416,K417,D420,Y421,Y453,L455,F456,R457,K458,N460,Y473,A475,G476,S477,F486,N487,Y489,Y495,N501,Y505 |
| 1087268 | Y144,Y145,H146,K147,K150,W152,H245,R246,S247,Y248,L249 |
| 1125015 | A372,F374,C379 |
| 1125016 | F374,S375,T376,F377,C379,F392,D427,E516 |
| 1181324 | L455,A475,G502 |
| 1181325 | Y449,Y453,L455,F456,E484,G485,F486,Y489,F490,L492,Q493,S494 |
| 1307796 | Y369,S375,F377,K378,C379,Y380,G381,V382,S383,P384,T385,K386,F392,P412,G413,D427,D428,F429,L517 |
| 1309150 | Y369,N370,F374,S375,T376,F377,K378,C379,Y380,G381,V382,S383,P384,T385,K386,L390,R408,D428,T430,L517,L518 |
| 1310987 | N334,L335,P337,G339,E340,N343,A344,T345,R346,K356,R357,S359,N360,C361,L441 |
| 1310988 | G446,Y449,Y453,L455,F456,A475,G476,S477,T478,G485,F486,N487,Y489,Q493,Y495,Q498,N501,Y505 |
| 1310989 | R346,F347,S349,Y351,K444,G446,G447,N448,Y449,N450,Y451,L452,T470,E484,F490,L492,Q493,S494 |
| 1311114 | L455,Y473,A475,G476,S477,E484,G485,F486,N487,C488,Y489 |
| 1311243 | G446,Y449,N481,G482,V483,E484,G485,F486,F490,S494 |
| 1311244 | K444,G446,Y449,N450,L452,N481,G482,V483,E484,G485,F490 |
| 1311245 | N334,L335,P337,G339,E340,N343,A344,T345,R346,K356,R357,S359,C361,L441 |
| 1311246 | R403,D405,T415,G416,K417,D420,Y421,Y453,L455,F456,R457,K458,N460,Y473,Q474,A475,G476,S477,T478,F486,N487,Y489,Q493,N501,Y505 |
| 1311247 | R403,T415,G416,K417,D420,Y421,Y453,L455,F456,R457,K458,N460,Y473,A475,G476,S477,F486,N487,Y489,Q493,S494,Y495,G496,Q498,T500,N501,G502,V503,Y505 |
| 1311248 | R403,T415,G416,K417,D420,Y421,Y453,L455,R457,K458,S459,N460,Y473,Q474,A475,G476,S477,F486,N487,Y489,Q493,Q498,T500,N501,G502,V503,Y505 |
| 1311249 | R403,V445,G446,Y449,Y453,L455,F456,N487,Y489,Q493,Y495,G496,Q498,P499,T500,N501,G502,Y505 |
| 1311250 | T415,G416,K417,D420,Y421,Y453,L455,F456,R457,K458,S459,N460,Y473,Q474,A475,G476,S477,F486,N487,Y489,Q493,S494,Y495,G496,Q498,T500,N501,G502,Y505 |
| 1311252 | Y369,N370,F377,K378,C379,Y380,G381,V382,S383,P384,T385,K386,N388,L390,F392,P412,Q414,D427,D428,F429,T430,L517 |
| 1311253 | Y369,N370,S371,A372,F374,S375,T376,F377,K378,C379,S383,P384,T385,R408,Q414 |
| 1314084 | D420,A475,N487 |
| 1314085 | E484,F490 |
| 1314086 | F486,N487 |
| 1314089 | S443,V445,G446,G447,Y449,G496,Q498,P499,T500 |
| 1314092 | T376,K378,R408,K417 |
| 1334439 | R346,K444,G446,G447,N448,Y449,N450,L452,V483,E484,G485,F490,S494 |
| 1334440 | R403,T415,G416,K417,D420,Y421,Y453,L455,F456,R457,K458,S459,N460,Y473,Q474,A475,G476,S477,F486,N487,Y489,Q493,G502,Y505 |
| 1334441 | V445,G446,Y449,F456,T478,N481,V483,E484,G485,F486,N487,Y489,F490,L492,Q493,S494,Q498,T500 |
| 1334512 | A123,G142,Y144,F157,R158,N164 |
| 1334513 | F140,Y144 |
| 1334676 | K417,E484,N501,R683 |
| 1334681 | N440 |
| 1334684 | E484,F490,Q493 |
| 1334685 | E484,Q493 |
| 1334686 | R346,N440 |
| 1335408 | R403,D405,T415,G416,K417,D420,Y421,Y453,L455,F456,R457,K458,N460,Y473,A475,G476,F486,N487,Y489,Q493,S494,Y495,G496,Q498,T500,N501,G502,G504,Y505 |
| 1335914 | E484 |
| 1335916 | F377 |
| 1335917 | F377,E516 |
| 1335918 | F377,K386,L390 |
| 1335919 | F377,T385,L390 |
| 1335920 | F377,Y508,E516 |
| 1335921 | F486 |
| 1335922 | F490 |
| 1335923 | G446 |
| 1335926 | G476,N501,Y508 |
| 1335930 | K444 |
| 1335934 | L441 |
| 1335935 | L452 |
| 1335936 | N354,F377,I468 |
| 1335937 | N354,K356,I468 |
| 1335938 | N450 |
| 1335940 | P499 |
| 1335943 | R403,K417,Y449,N450,L452,Y453,L455,F456,E484,G485,F486,Y489,F490,L492,Q493,S494,Y495,Y505 |
| 1335944 | S477 |
| 1335946 | S477,S514 |
| 1335949 | S494 |
| 1335950 | T345 |
| 1335952 | T478 |
| 1335954 | V341,F377,N501,V503,Y508,E516 |
| 1335955 | V503 |
| 1335957 | R403,R408,T415,G416,K417,D420,Y421,Y453,L455,F456,R457,K458,N460,Y473,Q474,A475,G476,F486,N487,Y489,Q493,S494,Y495,G496,Q498,T500,N501,G502,Y505 |
| 1335958 | R403,T415,G416,K417,D420,Y421,Y453,L455,F456,R457,K458,N460,Y473,A475,G476,F486,N487,Y489,Q493,S494,Y495,G496,Q498,T500,N501,G502,Y505 |
| 1335959 | R403,T415,G416,K417,D420,Y421,Y453,L455,F456,R457,K458,N460,Y473,A475,G476,S477,F486,N487,Y489,Q493,Q498,T500,N501,G502,Y505 |
| 1335960 | R403,T415,G416,K417,D420,Y421,Y453,L455,F456,R457,K458,N460,Y473,Q474,A475,G476,F486,N487,Y489,Q493,S494,Y495,G496,Q498,T500,N501,G502,Y505 |
| 1336251 | E484 |
| 1336252 | F456 |
| 1336253 | F490 |
| 1336254 | G446 |
| 1336256 | G485 |
| 1336263 | P384 |
| 1336265 | R403,D405,E406,R408,Q409,T415,G416,K417,D420,Y421,L455,F456,R457,K458,N460,Y473,Q474,A475,G476,S477,F486,N487,Y489,Q493,Y495,G502,Y505 |
| 1336267 | S494 |
| 1336532 | DISTEIYQAGSTPCNGVEGFNCYFPLQSYGFQPTNGVGYQPYRVVVL |
| 1337002 | IYQAGSTPCNGVEGFNCYFPLQSY |
| 1338206 | C136,N137,D138,P139,F140,L141,G142,V143,T307,V308,E309,K310,G311,I312,Y313,Q314,T315,S316,N317,F318,P621,V622,A623,I624,H625,A626,D627,Q628,L629,T630,P631,T632,W633,R634,V635,Y636 |
| 1338207 | C136,N137,D138,P139,F140,L141,G142,V143,Y144,L242,L244,H245,R246,S247,Y248,L249,T250,P251,G252,D253,S254,S255,S256,G257,W258,T259,A260,G261,A262,A263,A264,Y265 |
| 1338208 | C136,N137,D138,P139,F140,L141,G142,V143,Y144,V171,S172,Q173,P174,F175,L176,M177,D178,L179,L242,A243,L244,H245,R246,S247,Y248,L249,T250,P251,G252,D253,S254,S255,S256,G257,W258,T259,A260,G261,A262,A263,A264 |
| 1338212 | R403,D405,T415,G416,K417,D420,Y421,L455,F456,R457,K458,S459,N460,Y473,Q474,A475,G476,S477,F486,N487,Y489,N501,G502,Y505 |
| 1338213 | R403,T415,G416,K417,D420,Y421,Y453,L455,R457,K458,N460,Y473,A475,G476,S477,F486,N487,Y489,Q493,S494,Y495,G496,Q498,T500,N501,G502,V503,Y505 |
| 1338214 | V433,I434,A435,W436,N437,S438,N439,N440,L441,D442,S443,K444,V445,G446,G447,N448,Y449,N450,Y451,L452,Y453,R454,L455,G496,F497,Q498,P499,T500,N501,G502,V503,G504,Y505,Q506,P507,Y508,R509,V510,V511,V512,L513 |
| 1338215 | Y351,Y449,L455,T470,N481,G482,V483,E484,G485,F486,C488,Y489,F490,L492,Q493,S494 |
| 1338222 | Y369,S371,F377,K378,C379,Y380,G381,V382,S383,P384,T385,R408,P412,G413,Q414,T415,G416,D427,D428,F429 |
| 1338776 | T345,N439,N440,S443,K444,V445,G446,G447,N450,Q498,P499,T500,Q506 |
| 1338777 | Y351,K444,V445,G446,G447,N448,Y449,N450,L452,T470,E484,F490,L492,Q493,S494,Q498 |
| 1338778 | D405,K417,D420,L455,F456,N460,I472,Y473,A475,G476,F486,N487,Y489,G504 |
| 1338779 | G446,G447,Y449,F456,T470,V483,E484,G485,F486,C488,Y489,F490,P491,L492,Q493,S494,Q498 |
| 1338780 | K417 |
| 1338781 | K417,L455,F456,T470,E471,I472 |
| 1338782 | K444,V445,G446 |
| 1338783 | K444,V445,G446,L452,L455,F456,T470,E471,I472,S494 |
| 1338785 | L455,F456,S494,N501 |
| 1338786 | L455,F456,T470,E471,I472 |
| 1338787 | N501 |
| 1338788 | T470,E471,I472 |
| 1338789 | T470,E471,I472,N501 |
| 1338978 | K147,E484,N501 |
| 1346701 | N343,A344,T345,R346,S373,W436,N437,N440,L441,S443,K444,V445,N448,N450,R509 |
| 1346704 | Y369,N370,S371,F377,K378,C379,Y380,G381,V382,S383,P384,T385,R408,P412,G413,Q414,T415,G416,D427,D428,F429 |
| 1346803 | F140,G142,V143,Y145,H146,N148,N149,W152,E154,F157,A243,L244,H245 |
| 1346804 | S12,C15,L18,T19,C136,G142,H146,K147,N149,R246 |
| 1346805 | V16,N17,F140,G142,V143,Y144,Y145,H146,K147,N148,W152,E154,E156,R158,L244,H245,R246,L249,P251 |
| 1346806 | V16,N17,T19,Y144,R246,S247,Y248,T250,P251,G252,D253,S254,S255,S256,G257 |
| 1346807 | V16,N17,T20,Y144,Y145,H146,K147,N148,S155,R158,R246,L249,T250,P251,G252,D253 |
| 1346808 | Y351,G446,Y449,N450,L452,F456,T470,T478,P479,C480,N481,G482,V483,E484,G485,F486,N487,C488,Y489,F490,P491,L492,Q493,S494 |
| 1346809 | Y369,N370,S371,A372,F374,S375,T376,F377,K378,C379,S383,P384,D405,R408,Q409,Q414,T415,G416,N501,V503,G504,Y505 |
| 1346810 | Y449,L452,T470,E471,I472,N481,G482,V483,E484,G485,F486,F490,L492,Q493,S494 |
| 1346813 | K417,E484,N501 |
| 1346815 | L452,L455,F456,I472,N481,G482,V483,E484,G485,F486,Y489,F490 |
| 1346816 | L455,F456,K458,Y473,A475,G476,S477,T478,G485,F486,N487,Y489,Q493 |
| 1346817 | L455,K458,Y473,A475,G476,S477,T478,G485,F486,N487,C488,Y489,Q493 |
| 1346818 | N334,L335,P337,G339,E340,N343,A344,T345,K356,R357,S359,C361,N440,L441 |
| 1346820 | Q14,Y144,Y145,H146,K147,F157,G252,D253 |
| 1346821 | R403,D405,E406,R408,Q409,T415,G416,K417,D420,Y421,Y453,L455,F456,R457,K458,N460,Y473,Q474,A475,G476,S477,F486,N487,Y489,Y495,G496,Q498,T500,N501,G502,V503,Y505 |
| 1346822 | R403,D405,R408,T415,G416,K417,D420,Y421,Y453,L455,R457,K458,N460,Y473,Q474,A475,G476,S477,F486,N487,Y489,Q493,Y495,G496,Q498,T500,N501,G502,Y505 |
| 1346823 | R403,E406,Q409,T415,G416,K417,D420,Y421,Y453,L455,F456,R457,K458,N460,Y473,Q474,A475,G476,F486,N487,Y489,Q493,S494,Y495,G496,Q498,T500,N501,G502,Y505 |
| 1346824 | R403,K417,Y453,L455,F456,E484,G485,F486,N487,C488,Y489,Q493,N501,G502,Y505 |
| 1346825 | R403,T415,G416,K417,D420,Y421,Y453,L455,F456,R457,K458,S459,N460,Y473,Q474,A475,G476,S477,F486,N487,Y489,Y495,G496,Q498,T500,N501,G502,Y505 |
| 1346826 | W353,N354,R355,K356,R357,S359,N360,N394,Y396,P426,D428,K462,P463,F464,E465,R466,I468,E516,L518,H519,A520,T523 |
| 1346827 | Y449,L455,F456,V483,E484,G485,F486,Y489,F490,L492,Q493,S494 |
| 1346828 | Y473,A475,T478,F486,N487 |
| 1347684 | D614 |
| 1347685 | G446,N448,Y449,L452,E484,G485,F486,N487,Y489,F490,L492,Q493,S494 |
| 1347686 | R403,D405,E406,R408,T415,G416,K417,D420,Y421,Y449,L455,F456,R457,K458,N460,Y473,Q474,A475,G476,S477,N487,Y489,Q493,S494,G496,T500,N501,G502,V503,Y505 |
| 1347687 | R403,D405,R408,Q409,T415,G416,K417,Y421,Y449,Y453,L455,F456,G485,F486,N487,Y489,Q493,S494,Y495,G496,N501,Y505 |
| 1347688 | R403,E406,R408,Q409,G416,K417,Y449,Y453,L455,F456,F486,N487,Y489,Q493,S494,Y495,G496,Q498,T500,N501,G502,Y505 |
| 1347689 | R403,K417,Y449,L452,Y453,L455,F456,E484,G485,F486,C488,Y489,F490,L492,Q493,Y505 |
| 1347690 | R403,R408,Q409,Q414,T415,G416,K417,D420,Y421,G446,Y449,F456,A475,G476,S477,F486,N487,Y489,Q493,S494,G496,Q498,N501,Y505 |
| 1347691 | R403,T415,G416,K417,D420,Y421,L455,F456,R457,N460,Y473,A475,G476,F486,N487,Y489,Q493,G496,T500,N501,G502,Y505 |
| 1347692 | T415,G416,K417,D420,Y421,L455,F456,R457,K458,N460,Y473,Q474,A475,G476,S477,F486,N487,G496,Y505 |
| 1347693 | V483,E484,F486,Y489 |
| 1347694 | Y144 |
| 1347695 | Y421,F456,R457,Y473,A475,G476,S477,E484,G485,F486,N487,Y489,Q493 |
| 1347901 | K417,E484 |
| 1347902 | K417,N501 |
| 1347903 | L18,D80,L242,A243,L244,R246 |
| 1347904 | L18,Y144,L242,A243,L244,N501 |
| 1347905 | L18,Y144,L242,A243,L244,R246,E484,N501,A701,T716 |
| 1347906 | L242,A243,L244,E484 |
| 1347907 | S982 |
| 1347908 | Y144,L242,A243,L244,R246 |
| 1347909 | Y144,L242,A243,L244,R246,A570 |
| 1347910 | Q14,C15,V16,N17,T19,G142,V143,Y144,K147,E156,R158,L244,H245,R246,S247,Y248,L249,T250,P251,G252,S256 |
| 1347911 | Q14,C15,Y144,H146,K147,E154,E156,R158,R246,Y248,L249,T250,P251,D253,S254 |
| 1347912 | Q14,N17,L18,T76,K77,V143 |
| 1347913 | Q14,Y144,H146,K147,N148,N149,W152,M153,E154,E156,F157,R158,R246,Y248,L249,P251,G252,D253 |
| 1347914 | Y144,Y145,H146,K147,K150,W152,H245,R246,Y248,L249,T250,P251,G252,S254,S255,S256 |
| 1347915 | Y144,Y145,H146,K147,N148,K150,R246,S247,Y248,L249,T250,P251,G252,D253,S254 |
| 1379077 | DSFKEELDKYFKNHTS |
| 1386752 | PLQPELDSFKEELDKYFKNHTSPDV |
| 1391788 | G446,Y449,L452,T478,V483,E484,G485,F486,N487,Y489,F490,L492,Q493,S494,G496,Q498 |
| 1391789 | R403,D405,R408,T415,G416,K417,D420,Y421,L455,F456,R457,K458,N460,Y473,Q474,A475,G476,S477,F486,N487,Y489,Q493,T500,N501,G502,Y505 |
| 1391791 | W353,R355,R357,Y396,P426,D427,D428,F429,K462,P463,F464,R466,S514,E516,L518,H519,A520,P521 |
| 1391792 | Y144,W152,R246,Y248 |
| 1391793 | Y144,Y145,H146,K147,R246,S247,Y248,L249,T250,P251,G252,S255 |
| 1391794 | Y145,K147,W152,Y248 |
| 1391795 | Y369,N370,S371,A372,F374,S375,T376,F377,K378,C379,Y380,V382,S383,P384,T385,G404,D405,R408,T500,N501,G502,V503,G504,Q506 |
| 1391796 | Y449,L455,F456,V483,E484,G485,F486,Y489,F490,Q493,S494 |
| 1391797 | K444,G447 |
| 1391798 | N487 |
| 1391998 | K304,S371,N388 |
| 1391999 | K417,E484,N501 |
| 1392000 | N440 |
| 1392001 | R403,D405,A419,G502,G504,Y505,R509 |
| 1392002 | S71,K97,S98,T124,Y145,H146,K147,K150,S151,W152,E180,G181,K182,Q183,N185,V213,H245,S247,Y248,L249,T259,A260,A262 |
| 1392550 | F374,S375,T376,F377,K378,C379,Y380,G381,V382,S383,P384,T385,K386,R408,N437,V503,G504,Y508 |
| 1392552 | R346,Y351,K444,Y449,N450,L452,T470,I472,N481,G482,V483,E484,F490,L492,S494 |
| 1392553 | R403,T415,G416,K417,D420,Y421,Y453,L455,F456,R457,K458,N460,Y473,Q474,A475,G476,S477,F486,N487,Y489,Q493,S494,Y495,G496,Q498,T500,N501,G502,Y505 |
| 1392570 | S477 |
| 1392577 | N439,N440,S443,K444,V445,G446,G447,Y449,N450,S494,P499,T500,Q506 |
| 1392578 | R403,T415,G416,K417,D420,Y421,Y453,L455,F456,R457,K458,N460,Y473,Q474,A475,G476,S477,F486,N487,Y489,Q493,Y505 |
| 1392579 | T415,Y421,A475,G476,N487,S494,G502 |
| 1392580 | Y369,N370,F374,S375,T376,F377,K378,C379,Y380,G381,V382,S383,P384,T385,K386,F429,T430,F515,L517 |
| 1393655 | E484,N501 |
| 1393656 | K417 |
| 1393657 | K417,E484,N501 |
| 1393862 | KEELDKYFKNHTSPDVD |
| 1394090 | TEIYQAGSTPCNGVEGF |
| 1397231 | R346,N439,N440,S443,K444,V445,G446,G447,N450,Q498,P499,T500,N501,G502,Q506 |
| 1397232 | D405,K417,L455,F456,A475,G476,S477,E484,G485,F486,N487,Y489 |
| 1397518 | F456,Y473,A475,G476,S477,T478,V483,E484,G485,F486,N487,C488,Y489 |
| 1397519 | K417,N501 |
| 1397520 | L242,A243,L244 |
| 1397521 | N501 |
| 1397523 | R346,K444,Y449,N450,L452,I472,N481,G482,V483,E484,F490,L492 |
| 1397524 | R403,D405,T415,G416,K417,D420,Y421,L455,F456,R457,K458,N460,Y473,A475,G476,S477,E484,F486,N487,Y489,F490,Q493,S494,G496,Q498,N501,G502,Y505 |
| 1397525 | R403,D405,T415,G416,K417,D420,Y421,Y453,L455,F456,R457,K458,S459,N460,Y473,A475,G476,S477,F486,N487,Y489,Q493,G496,Q498,T500,N501,G502,Y505 |
| 1397526 | R403,T415,K417,D420,Y421,L455,R457,K458,N460,Y473,Q474,A475,G476,S477,T478,F486,N487,Y489,N501,G502,Y505 |
| 1397529 | E484 |
| 1397530 | N501 |
| 1398136 | A372,K378 |
| 1398141 | Q493 |
| 1461830 | KRSFIEDLLFNK |
| 1539320 | K417,L455,F456,Y473,Q474,A475,G476,S477,T478,C480,E484,G485,F486,C488,Y489,Q493 |
| 1539321 | K444,G446,S494,N450 |
| 1539322 | L455,F456,A475,G476,S477,T478,E484,F486,N487,Y489 |
| 1539325 | E340,V341,N343,T345,R346,F347,A348,Y351,N354,K356,L441,Y449,N450,R466,I468,T470,N481,G482,V483,E484,F490 |
| 1539326 | Q14,Y144,Y145,H146,K147,M153,F157,R158,R246,Y248,L249,T250,P251,G252,D253,S254 |
| 1556269 | Y351,K444,G446,Y449,N450,L452,T470,V483,E484,G485,F486,N487,C488,Y489,F490,L492,Q493,S494,Q498 |
| 1556270 | Y369,A372,S373,F374,S375,T376,F377,K378,C379,P384,R408,N437,G502,V503,Q506,Y508 |
| 1594752 | I472,E484,F486,N487,Y489,F490 |
| 1594753 | K417,L455,F456,Y473,I472,A475,E484,F486,N487,Y489,Q493 |
| 1594754 | K417,Y449,L455,F456,Y489,G496,Q498,T500,N501 |
| 1594755 | L455,Y473,A475,G476,S477,T478,E484,G485,F486,N487,C488,Y489,Q493 |
| 1594756 | N331,I332,T333,N334,L335,P337,G339,E340,N343,A344,T345,R346,K356,R357,S359,C361,L441 |
| 1594757 | S443,V445,G446,P499 |
| 1594758 | Y351,Y449,L452,F456,T470,G482,E484,G485,F486,Y489,F490,L492,S494 |
| 1594759 | Y369,S371,A372,F374,S375,T376,F377,K378,C379,V382,S383,P384,R403,G404,D405,R408,N501,G502,V503,G504,Y505,Y508 |
| 1594760 | Y369,S375,F377,K378,C379,Y380,G381,V382,S383,P384,T385,K386,L390,C391,F392,P412,D427,D428,F429,L517,K528 |
| 1594761 | Y449,F456,I472,Y473,N481,V483,E484,G485,F486,F490 |
| 1594762 | Y449,N450,L452,E484,F490,S494 |
| 1594765 | D614 |
| 1594766 | E484 |
| 1594767 | F140 |
| 1594768 | F140,E484 |
| 1595221 | SFKEELDKYF |
| 1596865 | S13,W152 |
| 1596867 | E484,S494,N501 |
| 1597448 | A475 |
| 1597449 | A831 |
| 1597450 | D614,I472 |
| 1597452 | F490 |
| 1597453 | H519 |
| 1597454 | L452 |
| 1597455 | N234 |
| 1597456 | N439 |
| 1597459 | V483 |
| 1597461 | E337,T342,R343,F344,A345,Y348,N351,K353,Y446,N447,L449,R463,I465,T467,V480,F487 |
| 1597462 | E337,T342,R343,Y348,N351,R352,K353,R354,Y446,N447,L449,R463,I465,S466,T467,E468,G479,F487,L489 |
| 1597463 | L452,F453,Y470,A472,G473,S474,T475,V480,G482,F483,N484,C485,Y486,Q490 |
| 1597464 | R343,N436,N437,L438,D439,S440,K441,V442,G443,G444,P496,T497 |
| 1597465 | R400,D402,E403,R405,Q406,T412,G413,N414,D417,Y418,L452,F453,R454,K455,N457,Y470,A472,G473,S474,T475,F483,N484,Y486,Q490,Y498,Y502 |
| 1597466 | T342,R343,N436,N437,L438,K441,V442,G443,G444,N447,Q495,P496,T497 |
| 1597467 | T342,R343,Y348,L438,K441,V442,G444,Y446,N447,L449,I465,T467,G479,K481,F487,L489,S491 |
| 1597468 | T412,G413,N414,D417,Y418,Y446,L452,F453,T475,P476,K481,G482,F483,N484,Y486,Q490,S491 |
| 1597629 | G339,E340,N343,A344,T345,R346,N437,N440,L441,D442,S443,K444,V445,N448,Y451,P499,T500 |
| 1597630 | G485,F486 |
| 1597631 | R346,K444,G446 |
| 1597632 | R403,T415,G416,K417,D420,Y421,L455,F456,R457,K458,N460,Y473,A475,G476,S477,F486,N487,Y489,Q493,Y495,T500,N501,G502,V503,Y505 |
| 1597633 | R403,T415,G416,K417,D420,Y421,Y453,L455,F456,R457,K458,N460,Y473,A475,G476,S477,F486,N487,Y489,Q493,T500,N501,G502,G504,Y505 |
| 1597880 | Y369,S371,A372,F374,S375,F377,K378,C379,Y380,G381,V382,S383,P384,T385,V407,R408,P412,G413,D427,D428,F429,T430,V503,G504 |
| 1597881 | Y369,S371,F377,K378,C379,Y380,G381,V382,S383,P384,T385,R408,P412,G413,Q414,T415,G416,D427,D428,F429,T430 |
| 1625483 | F342,T345,R346,L368,S373,F374,W436,N437,N440,L441,K444,V445 |
| 1625484 | T345,R346,N439,N440,L441,S443,K444,V445,G446,G447,N450,Q498,P499,T500,Q506 |
| 1633579 | Y369,S371,A372,F377,K378,C379,Y380,G381,V382,S383,P384,R408,P412,G413,Q414,T415,D420,D427,N460 |
| 1642855 | A348,N450,L452 |
| 1642856 | F456,N487 |
| 1642857 | G476,T478,G485,F486,N487 |
| 1642858 | K417,E484,N487,N501 |
| 1642859 | K417,F456,N460,A475,N487 |
| 1642860 | K417,F456,N487 |
| 1642861 | K417,L452,F490,N501 |
| 1642862 | L452,E484 |
| 1642863 | N343,T345,R346,W436 |
| 1642865 | R346,N450,L452,E484,F490 |
| 1642866 | R403,R408,K417,N501,Y505 |
| 1642867 | Y449,L452,F490 |
| 1642869 | E484,F486 |
| 1642870 | E484,N501 |
| 1642871 | H69,V70,Y144,L242,A243,L244,G261 |
| 1642872 | K417 |
| 1642873 | K417,E484 |
| 1642874 | K417,E484,F486 |
| 1642876 | K417,N439,Y453,E484 |
| 1642877 | K417,N501 |
| 1642878 | K417,Y453,S477,E484,F486 |
| 1642879 | L242,A243,L244 |
| 1642880 | N439,E484,N501 |
| 1642883 | N439,Y453,E484 |
| 1642884 | N439,Y453,T478,E484,N501 |
| 1642885 | S477,T478,F486 |
| 1642887 | V367,N439 |
| 1642888 | V367,N439,Y453 |
| 1642889 | W353,N354,R355,R357,Y449,N450,L452,R457,P463,F464,E465,R466,D467,I468,S469,T470,I472,G482,E484,F490,L492 |
| 1642890 | Y144,L242,A243,L244 |
| 1642891 | Y144,L242,A243,L244,G261 |
| 1642892 | Y144,Y145,H146,K147,K150,W152,R246,S247,Y248,L249 |
| 1711622 | K417,Y421,L455,F456,R457,K458,Y473,Q474,A475,G476,T478,P479,C480,V483,E484,G485,F486,N487,C488,Y489,F490,Q493 |
| 1711630 | Y145,K150,W152 |
| 1711631 | Y351,A352,W353,N360,L368,A419,V433,Y449,N450,D467,C480,E484,C488,F490,S494,R509 |
| 1711677 | N354,R355,R357,D428,L461,K462,P463,F464,E465,R466 |
| 1852152 | E406 |
| 1852153 | F486 |
| 1852155 | Q493 |
| 1852425 | L455,F456,A475,G476,S477,T478,P479,E484,G485,F486,N487,Y489,Q493 |
| 1860777 | E484,G485 |
| 1860778 | K417,Y453,L455,F456,Y473,A475,T478,E484,G485,F486,N487,Y489,Q493 |
| 1860779 | K444,G446,Y449,N450,L452,V483,E484,G485,F486,Y489,F490,L492,Q493,S494,Y495,G496,Q498,Y505 |
| 1860780 | Q14,V16,Y144,K147,R246,Y248,P251,G252,D253 |
| 1860781 | R355,R457,S459,K462,P463,F464,E465,R466,D467,I468,S469,E471,Q474,P479,C480,N481,G482,L518 |
| 1860782 | R403,K417,Y449,N501,Y505 |
| 1860783 | T415,G416,K417,D420,Y421,L455,F456,R457,K458,N460,Y473,Q474,A475,G476,S477,F486,N487,Y489,Q493,Y505 |
| 1860784 | W353,R355,Y396,R457,K462,P463,F464,E465,R466,D467,I468,E516,L518 |
| 1860785 | Y144,Y145,H146,K147,K150,W152,Y248 |
| 1860786 | Y145,H146,K147,R246,Y248,P251,G261 |
| 1860787 | Y145,K147,W152 |
| 1860788 | Y145,K147,Y248 |
| 1860789 | Y369,N370,S371,A372,F374,S375,T376,F377,K378,C379,P384,T385,N388 |
| 1860901 | K417,Y449,L452,L455,F456,E484,G485,F486,N487,C488,Y489,F490,L492,Q493,S494,G496,Q498,N501,G502,Y505 |
| 1860902 | A152,S154,T155,P156,C157,V160,K161,G162,F163,N164,C165,Y166 |
| 1860903 | F133,K161,G162,F163,Y166,Q170 |
| 1860904 | G123,Y126,Q170,S171,Y172,Q175,P176,T177,Y178,G179,Y182 |
| 1860905 | G16,E17,N20,A21,T22,R23,S50,F51,W113,N117,L118,D119,S120,K121,V122,N125,N127,Y128 |
| 1860906 | N117,S120,K121,V122,G123,Q175,P176,T177,Y178,G179,Y182 |
| 1860907 | R80,D82,R85,D97,Y98,Y130,L132,F133,R134,Y150,F163,N164,Y166,Q170,S171,Y172,G173,Y178,G179,V180,Y182 |
| 1860909 | R80,R85,T92,D97,Y98,L132,F133,F163,N164,Y166,F167,Q170,S171,Y172,G173,T177,Y178,G179,Y182 |
| 1860911 | R80,T92,G93,N94,D97,Y98,L132,F133,R134,K135,S136,N137,Y150,Q151,A152,G153,S154,F163,N164,Y166,Q170,T177,Y178,G179,Y182 |
| 1860912 | S52,R80,G81,D82,R85,V180,G181,Y182,Y185 |
| 1860913 | T10,N11,L12,C13,P14,F15,G16,E17,F19,N20,T22,R23,V39,A40,D41,Y42,V44,L45,K205 |
| 1860914 | T10,N11,L12,C13,P14,F15,G16,E17,F19,N20,T22,V39,A40,D41,Y42,V44,L45 |
| 1860915 | T92,Y98,L132,F133,R134,K135,Y150,A152,G153,S154,T155,P156,V160,G162,F163,N164,Q170 |
| 1860916 | V122,G123,Y126,G173,Q175,P176,T177,Y178 |
| 1860917 | V122,G123,Y126,Q170,Q175,P176,T177,Y178,G179,Y182 |
| 1860919 | Y28,Y126,N127,L129,T147,I149,P156,C157,N158,G159,V160,K161,F163,F167,L169 |
| 1860920 | S13,Q14,C15,V16,N17,F18,T19,T76,F140,Y144,Y145,H146,K147,N148,M153,S155,E156,R158,L246,T247,P248 |
| 1862805 | A344,T345,R346,F347,A348,S349,Y351,A352,N440,K444,V445,G446,G447,N448,Y449,N450 |
| 1862806 | G485,F486,N487 |
| 1862807 | R403,T415,G416,D420,Y421,L455,F456,R457,K458,N460,Y473,Q474,A475,G476,S477,E484,F486,N487,Y489,F490,L492,Q493,N501,G502,Y505 |
| 1862808 | T345,R346,N440,L441,S443,K444,V445,N448,N450,Y451,P499,T500 |
| 1862813 | A123,T124,Y144,Y145,H146,K147,K150,S151,W152,M153 |
| 1862954 | N331,I332,T333,N334,L335,P337,G339,E340,N343,A344,T345,R346,K356,R357,S359,C361,L441 |
| 1862955 | Y351,Y449,L455,T470,N481,G482,V483,E484,G485,F486,C488,Y489,F490,L492,Q493,S494 |
| 1862956 | Y369,N370,S371,A372,S373,F374,S375,T376,F377,K378,C379,S383,P384,T385,N437 |
| 1864082 | L455,K458,A475,G476,S477,T478,V483,E484,F486,N487,Q493 |
| 1864083 | L455,K458,Y473,A475,G476,S477,T478,V483,E484,G485,F486,N487,Q493 |
| 1864085 | N343,T345,R346,N439,N440,L441,D442,S443,K444,V445,N448,Y451,P499,T500,R509 |
| 1864086 | N439,N440,L441,D442,S443,K444,V445,G446,G447,N448,Y449,N450,Y451,L452,Y453,R454,L455,F456,R457,K458,P499,T500,N501,G502,V503,G504,Y505,Q506,P507,Y508,R509,V510,V511,V512,L513,S514,F515,E516,L517,L518 |
| 1864087 | N99,R102,G103,W104,I119,N121,Y170,S172,Q173,P174,F175,L176,M177,D178,E180,K182,N188,R190,F192,H207,E224,L226 |
| 1864088 | R346,N439,N440,L441,S443,K444,V445,G446,G447,N448,Y449,N450,Q498,P499,T500 |
| 1864092 | Y369,N370,S371,A372,F374,F377,K378,C379,Y380,G381,V382,S383,P384,T385,K386,L390,F429,T430,F515,E516,L517 |
| 1870743 | F456,A475,G476,S477,T478,E484,G485,F486,N487,Y489 |
| 1870744 | E484,F490 |
| 1870745 | E484,Q493 |
| 1870746 | G446 |
| 1870747 | G476,F486 |
| 1870748 | K417,Q493 |
| 1870749 | K444 |
| 1870750 | K444,G446 |
| 1870751 | K444,G446,L452 |
| 1870752 | L452,E484 |
| 1870753 | L452,E484,F490 |
| 1870754 | L452,Q493 |
| 1870755 | R403,Q409,T415,G416,K417,D420,Y421,Y453,L455,F456,R457,K458,N460,Q474,F486,N487,Y489,Q493,S494,G496,N501,V503,Y505 |
| 1874115 | G443 |
| 1874136 | K441 |
| 1874139 | K481 |
| 1874148 | L449 |
| 1874162 | N414 |
| 1874243 | Y498 |
| 1945258 | L455,A475,G476,S477,T478,E484,G485,F486,N487,C488,Y489,Q493 |
| 1945259 | R346,N439,N440,L441,S443,K444,V445,G446,Y449,N450,L452,E484,F490,Q493,S494,P499 |
| 1945260 | R403,D405,E406,R408,T415,G416,K417,D420,Y421,Y449,Y453,L455,F456,R457,K458,N460,Y473,A475,G476,S477,F486,N487,Y489,Q493,S494,Y495,G496,Q498,T500,N501,G502,Y505 |
| 1945261 | R403,D405,T415,G416,K417,D420,Y421,Y453,L455,F456,R457,K458,N460,Y473,Q474,A475,G476,S477,F486,N487,Y489,Q493,G502,Y505 |
| 1945262 | R403,T415,G416,K417,D420,Y421,Y453,L455,F456,R457,K458,N460,Y473,A475,G476,S477,F486,N487,Y489,Q493,Y495,G496,Q498,T500,N501,G502,V503,Y505 |
| 1945263 | D402,R405,T412,G413,N414,D417,Y418,L452,F453,R454,K455,N457,Y470,A472,G473,F483,N484,Y486,Q490,Y498,G499,Y502 |
| 1945264 | L452,Y470,A472,G473,S474,T475,G482,F483,N484,C485,Y486,Q490 |
| 1957828 | I332,T333,N334,L335,C336,P337,G339,E340,V341,N343,A344,T345,R346,N354,K356,R357,I358,S359,N360,C361,N440,L441,R509 |
| 1957829 | K417,Y421,Y449,L452,L455,F456,A475,G476,T478,E484,G485,F486,N487,C488,Y489,F490,L492,Q493,S494,G496,Q498,N501,G502,Y505 |
| 1957830 | L455,F456,Q474,A475,G476,S477,T478,P479,E484,G485,F486,N487,C488,Y489,Q493 |
| 1957831 | R403,D405,E406,Q409,T415,G416,K417,D420,Y421,Y453,L455,F456,R457,K458,N460,Y473,A475,G476,S477,F486,N487,Y489,Q493,S494,Y495,G496,Q498,T500,N501,G502,V503,Y505 |
| 1957832 | R403,D405,E406,R408,Q409,T415,G416,K417,D420,Y421,Y453,L455,F456,R457,K458,S459,N460,Y473,Q474,A475,G476,S477,F486,N487,Y489,Q493,S494,Y495,T500,N501,G502,V503,G504,Y505 |
| 1957834 | R403,K417,G446,Y449,N450,L452,Y453,L455,F456,V483,E484,G485,F486,Y489,F490,L492,Q493,S494,Y495,Y505 |
| 1957837 | T345,R346,N439,N440,L441,S443,K444,V445,G446,G447,Y449,N450,L452,E484,F490,L492,Q493,S494,P499 |
| 1957840 | W353,R355,T393,N394,Y396,P426,D427,D428,F429,T430,K462,P463,F464,S514,F515,E516,L518,H519,A520,P521 |
| 1957842 | Y351,Y449,N450,L452,L455,F456,R457,T470,I472,N481,G482,V483,E484,G485,F486,N487,C488,Y489,F490,L492,Q493,S494,Y495,G496, |
| 1957843 | Y369,N370,S371,A372,S373,F374,S375,T376,F377,K378,C379,Y380,V382,S383,P384,T385,K386,L387,G404,D405,R408,Q409,G502,V503,G504,Q506,Y508 |
| 1972666 | L242,A243,L244 |
| 1972667 | R403,D405,E406,R408,T415,G416,K417,D420,Y421,Y449,Y453,L455,F456,R457,K458,N460,Y473,A475,G476,S477,F486,N487,Y489,Q493,S494,Y495,G496,Q498,T500,N501,G502,Y505 |
| 1972668 | R403,D405,T415,G416,K417,D420,Y421,Y453,L455,F456,R457,K458,N460,Y473,Q474,A475,G476,S477,F486,N487,Y489,Q493,G502,Y505 |
| 1972669 | R403,T415,G416,K417,D420,Y421,Y453,L455,F456,R457,K458,N460,Y473,A475,G476,S477,F486,N487,Y489,Q493,Y495,G496,Q498,T500,N501,G502,V503,Y505 |
| 1972671 | R400,T412,G413,N414,D417,Y418,Y450,L452,F453,R454,K455,N457,Y470,Q471,A472,G473,S474,F483,N484,Y486,Q490,T497,Y498,G499,Y502 |
| 1972731 | R403,D405,R408,T415,G416,K417,D420,Y421,Y453,L455,F456,R457,K458,N460,Y473,Q474,A475,G476,S477,F486,N487,Y489,Q493,N501,G502,Y505 |
| 1972934 | E340,A344,T345,R346,F347,A348,Y351,A352,N354,K356,Y449,N450,R466,I468,T470,N481,F490 |
| 1972935 | T345,R346,F347,A348,S349,Y351,N440,L441,D442,S443,K444,V445,N448,N450,Y451,R509 |
| 1972961 | DKYFKNHTSPDVDL |
| 2000935 | L452,L455,F456,I472,N481,G482,V483,E484,G485,F486,Y489,F490 |
| 2000936 | N331,I332,T333,N334,L335,P337,G339,E340,N343,A344,T345,R346,K356,R357,S359,C361,L441 |
| 2000937 | Q14,Y144,Y145,H146,K147,F157,G252,D253 |
| 2000940 | R403,D405,E406,R408,Q409,T415,G416,K417,D420,Y421,Y453,L455,F456,R457,K458,N460,Y473,Q474,A475,G476,S477,F486,N487,Y489,Y495,G496,Q498,T500,N501,G502,V503,Y505 |
| 2000941 | R403,D405,R408,T415,G416,K417,D420,Y421,Y453,L455,R457,K458,N460,Y473,Q474,A475,G476,S477,F486,N487,Y489,Q493,Y495,G496,Q498,T500,N501,G502,Y505 |
| 2000942 | R403,E406,Q409,T415,G416,K417,D420,Y421,Y453,L455,F456,R457,K458,N460,Y473,Q474,A475,G476,F486,N487,Y489,Q493,S494,Y495,G496,Q498,T500,N501,G502,Y505 |
| 2000943 | R403,K417,Y453,L455,F456,E484,G485,F486,N487,C488,Y489,Q493,N501,G502,Y505 |
| 2000944 | R403,T415,G416,K417,D420,Y421,Y453,L455,F456,R457,K458,S459,N460,Y473,Q474,A475,G476,S477,F486,N487,Y489,Y495,G496,Q498,T500,N501,G502,Y505 |
| 2000945 | Y351,Y449,L455,T470,N481,G482,V483,E484,G485,F486,C488,Y489,F490,L492,Q493,S494 |
| 2000946 | Y449,L455,F456,V483,E484,G485,F486,Y489,F490,L492,Q493,S494 |
| 2000948 | R403,D405,R408,T415,G416,K417,D420,Y421,L455,F456,R457,K458,N460,Y473,Q474,A475,G476,S477,F486,N487,Y489,Q493,T500,N501,G502,Y505 |
| 2000949 | T345,R346,Y351,N440,L441,D442,S443,K444,V445,G446,G447,N448,Y449,N450,L452,T470,F490,P499 |
| 2000951 | Y453,L455,Y473,A475,G476,S477,T478,G485,F486,N487,Y489,Q493 |
| 2001237 | PPLLTDEMIAQYTSA |
| 2060662 | L455,A475,G476,S477,T478,E484,G485,F486,N487,C488,Y489,Q493 |
| 2060663 | R346,N439,N440,L441,S443,K444,V445,G446,Y449,N450,L452,E484,F490,Q493,S494,P499 |
| 2060664 | R403,D405,R408,Q498,T500,N501,G502,V503,G504,Y505,Q506 |
| 2060665 | Y369,N370,S371,A372,F374,S375,T376,F377,K378,C379,S383,P384,D405,R408,Q409,Q414,T415,G416,N501,V503,G504,Y505 |
| 2060666 | Y369,N370,S371,A372,F374,S375,T376,F377,K378,C379,Y380,V382,S383,P384,T385,G404,D405,R408,T500,N501,G502,V503,G504,Q506 |
| 2060701 | D405,K417,D420,L455,F456,N460,I472,Y473,A475,G476,F486,N487,Y489,G504 |
| 2060704 | K417,L455,F456,Y473,A475,S477,T478,E484,G485,F486,N487,Y489,F490,L492,Q493 |
| 2060926 | Y369,N370,A372,F374,S375,T376,K378,C379,G404,D405,V407,R408,G502,V503,G504,Y508 |
| 2060928 | K417,L455,F456,Y473,Q474,A475,G476,S477,T478,G485,F486,N487,C488,Y489,Q493 |
| 2060929 | T345,R346,N439,N440,L441,D442,S443,K444,V445,N448,Y451,P499,T500 |
| 2060933 | T342,R343,N436,K437,L438,D439,S440,K441,V442,N445,N447,Y448,P496,T497 |
| 2061157 | F43,F175,L176,L226 |
| 2061158 | R102,Y145,K147,W152,R246,Y248,P251,G252 |
| 2061159 | Y366,A369,P370,F371,F372,T373,K375,Y377,S380,P381,T382,K383,G401,D402,V404,R405,I407,A408,V430,A432,V500,G501,Y505,V507 |
