# Supplementary material for: Immune targets to stop future SARS-CoV-2 variants
Source: Microbiol Spectr. 2023 Nov 15;11(6):e02892-23. doi: 10.1128/spectrum.02892-23 (PMC10714790; doi:10.1128/spectrum.02892-23)
Supplement: Supplemental material 3 — Amino acid sequences of T-cell epitopes. [file spectrum.02892-23-s0003.docx]

| **epitopes MHC-I** | | **epitopes MHC-II** | |
| --- | --- | --- | --- |
| **Epitope ID** | **Description** | **Epitope ID** | **Description** |
| 1332003 | FVFLVLLPL | 7868 | MFVFLVLLPLVSS |
| 1310705 | PAYTNSFTRGVYYPD | 18514 | PPAYTNSFTRGVYYP |
| 1072624 | SFTRGVYYPDKVFRS | 23293 | PAYTNSFTRGVYYPD |
| 1333812 | STQDLFLPF | 25754 | TNSFTRGVYYPDKVFRSS |
| 1310610 | LPFFSNVTWFHAIHV | 38990 | SFTRGVYYPDKVFRS |
| 1310701 | NVTWFHAIHVSGTNG | 39003 | TRGVYYPDKVFRSSV |
| 1087399 | RFDNPVLPF | 51112 | TRGVYYPDKVFRS |
| 2145131 | RFDNPVLPFNDGVYFAST | 52057 | VYYPDKVFRSSVLHS |
| 1321049 | LPFNDGVYF | 60024 | VYYPDKVFRSSVLHSTQD |
| 1615663 | PFNDGVYFASTEKSNIIR | 100428 | KVFRSSVLHSTQDLF |
| 1312627 | GVYFASTEK | 532384 | FRSSVLHSTQDLFLPFFS |
| 1074069 | YFASTEKSNIIRGWI | 1069137 | SSVLHSTQDLFLP |
| 1603889 | FASTEKSNIIRGWIFGTT | 1069290 | SVLHSTQDLFLPFFS |
| 1075071 | TEKSNIIRGW | 1069291 | HSTQDLFLPFFSN |
| 1310361 | EKSNIIRGWIFGTTL | 1069347 | LPFFSNVTWFHAIHV |
| 1614938 | NIIRGWIFGTTLDSKTQS | 1069350 | PFFSNVTWFHAIHVSGTN |
| 1310506 | IRGWIFGTTLDSKTQ | 1069378 | PFFSNVTWFHAIHVS |
| 1310930 | VVIKVCEFQFCNDPF | 1069445 | NVTWFHAIHVSGTNG |
| 1310306 | CEFQFCNDPFLGVYY | 1069550 | WFHAIHVSGTNGTKRFDN |
| 1888600 | FCNDPFLGVY | 1069816 | TKRFDNPVLPFNDGV |
| 1597047 | GVYHKNNK | 1069822 | RFDNPVLPFNDGVYFAST |
| 1312630 | GVYYHKNNK | 1070353 | FNDGVYFASTEKSNI |
| 1313211 | NKSWMESEFRVYSSA | 1070803 | YFASTEKSNIIRGWI |
| 1327695 | VYSSANNCTF | 1071133 | FASTEKSNIIRGWIFGTT |
| 1310820 | SSANNCTFEYVSQPF | 1071338 | EKSNIIRGWIFGTTL |
| 1313194 | NCTFEYVSQPFLMDL | 1071575 | NIIRGWIFGTTLDSKTQS |
| 1312410 | FEYVSQPFL | 1071580 | IRGWIFGTTLDSKTQ |
| 1316287 | EYVSQPFLM | 1071585 | NNATNVVIKVCEFQF |
| 1310922 | VSQPFLMDLEGKQGN | 1071586 | NVVIKVCEFQFCNDP |
| 1309110 | CTFEYVSQPFLMDLE | 1071788 | VVIKVCEFQFCNDPF |
| 1069822 | GKQGNFKNLREFVFK | 1071818 | VIKVCEFQFCNDPFLGVY |
| 1309113 | EFVFKNIDGYFKIYS | 1071978 | KVCEFQFCNDPFLGVYYHK |
| 1326409 | VFKNIDGYF | 1072541 | KVCEFQFCNDPFL |
| 1310664 | NIDGYFKIYSKHTPI | 1072624 | CEFQFCNDPFLGVYY |
| 1310401 | FKIYSKHTPINLVRD | 1072807 | FQFCNDPFLGVYYHK |
| 1309123 | KHTPINLVRDLPQGF | 1073276 | FQFCNDPFLGVYY |
| 1071585 | NLVRDLPQGFSALEP | 1073438 | FQFCNDPFLGVYYHKNNK |
| 1310487 | IGINITRFQTLLALH | 1073698 | QFCNDPFLGVYYHKNNKS |
| 1310865 | TRFQTLLALHRSYLT | 1073938 | CNDPFLGVYYHKNNK |
| 1312633 | GWTAGAAAYYVGYLQ | 1073956 | NKSWMESEFRVYSSA |
| 1327824 | WTAGAAAYY | 1074069 | SSANNCTFEYVSQPF |
| 2144756 | AGAAAYYVGYLQPRTFLL | 1074201 | SSANNCTFEYVSQPFLMDL |
| 1075131 | YYVGYLQPRTFLL | 1087507 | SANNCTFEYVSQPFLMDL |
| 1317916 | GYLQPRTFLL | 1087668 | NCTFEYVSQPFLMDL |
| 1547648 | GYLQPRTFL | 1087674 | NCTFEYVSQPFLM |
| 1309147 | YLQPRTFLL | 1087748 | CTFEYVSQPFLMDLE |
| 1874038 | YLQPRTFLLKYNE | 1087755 | CTFEYVSQPFLMD |
| 1310445 | GIYQTSNFRVQPTES | 1087780 | TFEYVSQPFLMDLE |
| 1319328 | IYQTSNFRV | 1087807 | EYVSQPFLMDLEGKQGNF |
| 1310814 | SNFRVQPTESIVRFP | 1309110 | VSQPFLMDLEGKQGN |
| 1313203 | NFRVQPTESIVRFPN | 1309113 | GKQGNFKNLREFVFK |
| 1310745 | QPTESIVRFPNITNL | 1309117 | FKNLREFVFKNIDGY |
| 1323461 | QPTESIVRF | 1309118 | LREFVFKNIDGYFKIYSKHTPINLVRD |
| 1323786 | RFPNITNLCPF | 1309120 | REFVFKNIDGYFKIYSKH |
| 1310312 | CPFGEVFNATRFASV | 1309123 | EFVFKNIDGYFKIYS |
| 1310886 | VFNATRFASVYAWNR | 1309132 | NIDGYFKIYSKHTPI |
| 1599614 | ATRFASVYAWNRKRISNC | 1309139 | IDGYFKIYSKHTPIN |
| 1310765 | RFASVYAWNRKRISN | 1309140 | IDGYFKIYSKHTPINLVR |
| 1325172 | SVYAWNRKR | 1309143 | IDGYFKIYSKHTP |
| 1327895 | YAWNRKRI | 1309144 | FKIYSKHTPINLVRD |
| 1310551 | KRISNCVADYSVLYN | 1309494 | YSKHTPINLVRDLPQGFS |
| 1313442 | RISNCVADYSVLYNS | 1309497 | SKHTPINLVRDLPQG |
| 1542531 | SNCVADYSVLYNSAS | 1309913 | KHTPINLVRDLPQGF |
| 7247 | CVADYSVLY | 1309940 | PINLVRDLPQGFSAL |
| 1310787 | SASFSTFKCYGVSPT | 1310253 | PINLVRDLPQGFS |
| 1599400 | ASFSTFKCYGVSPTKLND | 1310259 | NLVRDLPQGFSALEP |
| 1311170 | KCYGVSPTK | 1310281 | NLVRDLPQGFSALEPLVD |
| 1310948 | YADSFVIRGDEVRQI | 1310282 | IGINITRFQTLLALH |
| 1872111 | GQTGKIADYNYKL | 1310284 | GINITRFQTLLALHRSYL |
| 1597230 | NIADYNYKL | 1310294 | INITRFQTLLALH |
| 1319519 | KIADYNYKL | 1310304 | ITRFQTLLALHRSYL |
| 1597376 | TIADYNYKL | 1310306 | TRFQTLLALHRSYLT |
| 1601336 | DSKVGGNYNYLYRLFRKS | 1310309 | LLALHRSYLTPGDSS |
| 1309117 | GGNYNYLYRLFRKSN | 1310311 | PGDSSSGWTAGAAAY |
| 1313269 | NYNYLYRLF | 1310312 | GWTAGAAAYYVGYLQ |
| 1597243 | NYNYRYRLF | 1310326 | ENGTITDAVDCAL |
| 1087807 | YNYLYRLFRKSNLKPFER | 1310327 | ITDAVDCALDPLS |
| 1329862 | NYLYRLFRKSNLKPF | 1310330 | TDAVDCALDPLSETK |
| 1074201 | YLYRLFRKSNLKPFE | 1310337 | PLSETKCTLKSFTVEKGI |
| 1075031 | RLFRKSNLK | 1310345 | LSETKCTLKSFTVEK |
| 1604361 | FRKSNLKPFERDISTEIY | 1310360 | CTLKSFTVEKGIYQT |
| 1310589 | LKPFERDISTEIYQA | 1310361 | TLKSFTVEKGIYQTSNFR |
| 1319894 | KPFERDISTEI | 1310401 | KSFTVEKGIYQTSNFRVQ |
| 1310360 | EIYQAGSTPCNGVEG | 1310403 | FTVEKGIYQTSNFRV |
| 1314028 | YQAGSTPCNGVEGFN | 1310412 | GIYQTSNFRVQPTES |
| 1602300 | EGFNCYFPLQSYGFQPTN | 1310413 | YQTSNFRVQPTESIVRFPN |
| 1312542 | GFNCYFPLQSYGFQP | 1310415 | SNFRVQPTESIVRFP |
| 1310412 | FNCYFPLQSYGFQPT | 1310423 | SNFRVQPTESIVRFPNIT |
| 2150983 | YFPLRSYGF | 1310444 | NFRVQPTESIVRFPN |
| 2150984 | YFPLRSYSF | 1310445 | QPTESIVRFPNITNL |
| 1075121 | YFPLQSYGF | 1310457 | IVRFPNITNLCPFGE |
| 1313990 | YFPLQSYGFQPTNGV | 1310476 | CPFGEVFNATRFASV |
| 2150982 | YFPLQSYSF | 1310485 | FGEVFNATRFASVYA |
| 1310441 | GFQPTNGVGYQPYRV | 1310487 | VFNATRFASVYAWNR |
| 1087346 | FQPTNGVGY | 1310503 | ATRFASVYAWNRKRISNC |
| 1310747 | QPYRVVVLSFELLHA | 1310506 | TRFASVYAWNRKRIS |
| 1313517 | RVVVLSFELLHAPAT | 1310513 | TRFASVYAWNRKRISNCVA |
| 1309118 | GPKKSTNLVKNKCVN | 1310515 | TRFASVYAWNRKRISNCVADYSVLYNS |
| 1310671 | NKKFLPFQQFGRDIA | 1310528 | RFASVYAWNRKRISN |
| 1599765 | AVRDPQTLEILDITPCSF | 1310542 | SVYAWNRKR |
| 1310653 | NCTEVPVAIHADQLT | 1310548 | SVYAWNRKRISNC |
| 1324117 | RVYSTGSNVF | 1310550 | YAWNRKRISNCVADY |
| 1310978 | YSTGSNVFQTRAGCL | 1310551 | YAWNRKRISNCVADYSVL |
| 1310910 | VNNSYECDIPIGAGI | 1310555 | WNRKRISNCVADYSV |
| 1311590 | SPRRARSVA | 1310586 | WNRKRISNCVADYSVLYNS |
| 1309137 | SIIAYTMSL | 1310589 | KRISNCVADYSVLYN |
| 1310803 | SIIAYTMSLGAENSV | 1310592 | RISNCVADYSVLYNSASFS |
| 1310855 | TNFTISVTTEILPVS | 1310593 | RISNCVADYSVLY |
| 1317060 | FTISVTTEI | 1310610 | ISNCVADYSVLYNSASFS |
| 1310649 | MTKTSVDCTMYICGD | 1310614 | CVADYSVLYNSASFS |
| 1309139 | STECSNLLLQYGSFC | 1310618 | SASFSTFKCYGVSPT |
| 1071580 | NLLLQYGSFCTQLNR | 1310633 | TKLNDLCFTNVYADS |
| 1310863 | TQLNRALTGIAVEQD | 1310649 | GVSPTKLNDLCFTNV |
| 1615277 | NRALTGIAVEQDKNTQEV | 1310660 | TNVYADSFVIRGDEVRQI |
| 1310884 | VFAQVKQIYKTPPIK | 1310664 | YADSFVIRGDEVRQI |
| 1619782 | SKRSFIEDLLFNKVTLAD | 1310669 | FVIRGDEVRQIAPGQTGK |
| 1310796 | SFIEDLLFNKVTLAD | 1310671 | VIRGDEVRQIAPGQT |
| 37289 | LLFNKVTLA | 1310695 | VIRGDEVRQIAPG |
| 1314170 | ADAGFIKQY | 1310701 | PDDFTGCVIAWNSNN |
| 1310259 | AGFIKQYGDCLGDIA | 1310705 | FTGCVIAWNSNNLDSKVGG |
| 2145107 | PPLLTDEMIAQYTSALLA | 1310707 | LDSKVGGNYNYLYRLFRKS |
| 1310623 | LTDEMIAQY | 1310714 | DSKVGGNYNYLYRLFRKS |
| 1313153 | MIAQYTSAL | 1310725 | VGGNYNYLYRLFRKS |
| 18514 | GAALQIPFAMQMAYR | 1310733 | VGGNYNYLYRLFRKSNLKP |
| 1310503 | IPFAMQMAYRFNGIG | 1310739 | GGNYNYLYRLFRKSN |
| 1318829 | IPFAMQMAY | 1310745 | GNYNYLYRLFRKSNL |
| 1316310 | FAMQMAYRF | 1310747 | GNYNYLYRLFRKSNLKPFER |
| 1310927 | VTQNVLYENQKLIAN | 1310750 | YNYLYRLFRKSNLKPFER |
| 1310542 | KLIANQFNSAIGKIQ | 1310761 | YNYLYRLFRKSNLKP |
| 2801 | ALNTLVKQL | 1310765 | YNYLYRLFRKSNL |
| 69657 | VLNDILSRL | 1310787 | NYLYRLFRKSNLKPF |
| 54507 | RLDKVEAEV | 1310796 | YLYRLFRKSNLKPFE |
| 1073938 | VQIDRLITGRLQSLQ | 1310803 | FRKSNLKPFERDISTEIY |
| 22144 | GRLQSLQTY | 1310814 | LKPFERDISTEIYQA |
| 54725 | RLQSLQTYV | 1310820 | PFERDISTEIYQAGSTPC |
| 39003 | LQTYVTQQLIRAAEI | 1310825 | RDISTEIYQAGSTPC |
| 1310876 | TYVTQQLIRAAEIRA | 1310827 | STEIYQAGSTPCNGV |
| 1310739 | QLIRAAEIRASANLA | 1310834 | EIYQAGSTPCNGVEG |
| 1310294 | ATKMSECVLGQSKRV | 1310847 | NGVEGFNCYFPLQSY |
| 1072604 | SFPQSAPHGVVFLHV | 1310848 | GFNCYFPLQSYGF |
| 1324414 | SFPQSAPHGVVF | 1310850 | FNCYFPLQSYGFQPT |
| 1310281 | APHGVVFLHVTYVPA | 1310852 | NGVGYQPYRVVVLSF |
| 71663 | VVFLHVTYV | 1310854 | VGYQPYRVVVLSFELLHAPA |
| 1310885 | VFLHVTYVPAQEKNF | 1310855 | QPYRVVVLSFELLHA |
| 1310875 | TYVPAQEKNFTTAPA | 1310857 | RVVVLSFELLHAPATVCGP |
| 1310476 | HWFVTQRNFYEPQII | 1310863 | VVLSFELLHAPATVC |
| 1318219 | HWFVTQRNF | 1310865 | LSFELLHAPATVCGP |
| 1310750 | QRNFYEPQIITTDNT | 1310870 | GPKKSTNLVKNKCVN |
| 1069445 | EPQIITTDNTFVSGN | 1310871 | KSTNLVKNKCVNF |
| 71996 | VYDPLQPEL | 1310874 | TNLVKNKCVNFNFNG |
| 1310614 | LQPELDSFKEELDKY | 1310875 | NKCVNFNFNGLTGTG |
| 1312935 | KYEQYIKWPWYIWLG | 1310876 | FNFNGLTGTGVLTES |
| 1309144 | YEQYIKWPWYIWLGF | 1310877 | ESNKKFLPFQQFGRDIAD |
| 1311601 | YEQYIKWPW | 1310884 | NKKFLPFQQFGRDIA |
| 1310756 | QYIKWPWYI | 1310885 | KKFLPFQQFGRDI |
| 1311572 | KWPWYIWLGF | 1310886 | GRDIADTTDAVRDPQ |
| 1624516 | WPWYIWLGFIAGLIAIVM | 1310891 | DTTDAVRDPQTLEIL |
| 16156 | FIAGLIAIV | 1310902 | VRDPQTLEILDITPC |
| 57592 | SEPVLKGVKL | 1310910 | TLEILDITPCSFGGV |
|  |  | 1310916 | DITPCSFGGVSVITP |
|  |  | 1310922 | QDVNCTEVPVAIHADQLTP |
|  |  | 1310925 | PTWRVYSTGSNVFQT |
|  |  | 1310927 | YSTGSNVFQTRAGCL |
|  |  | 1310930 | NVFQTRAGCLIGAEH |
|  |  | 1310931 | NVFQTRAGCLIGAEHVNNS |
|  |  | 1310935 | VNNSYECDIPIGAGI |
|  |  | 1310947 | NNSYECDIPIGAGIC |
|  |  | 1310948 | NNSYECDIPIGAGICASYQ |
|  |  | 1310955 | NNSYECDIPIGAG |
|  |  | 1310978 | ECDIPIGAGICASYQ |
|  |  | 1312272 | PIGAGICASYQTQ |
|  |  | 1312282 | IGAGICASYQTQTNS |
|  |  | 1312421 | CASYQTQTNSPRRAR |
|  |  | 1312484 | PRRARSVASQSIIAY |
|  |  | 1312633 | SVASQSIIAYTMSLG |
|  |  | 1312689 | SIIAYTMSLGAENSV |
|  |  | 1312935 | TMSLGAENSVAYSNN |
|  |  | 1313008 | TNFTISVTTEILPVS |
|  |  | 1313083 | NFTISVTTEILPV |
|  |  | 1313154 | MTKTSVDCTMYICGD |
|  |  | 1313194 | VDCTMYICGDSTECS |
|  |  | 1313203 | VDCTMYICGDSTECSNLL |
|  |  | 1313211 | DCTMYICGDSTECSN |
|  |  | 1313230 | CGDSTECSNLLLQYGSFC |
|  |  | 1313262 | DSTECSNLLLQYGSF |
|  |  | 1313276 | STECSNLLLQYGSFC |
|  |  | 1313285 | TECSNLLLQYGSFCTQL |
|  |  | 1313359 | CSNLLLQYGSFCTQL |
|  |  | 1313363 | SNLLLQYGSFCTQLNRAL |
|  |  | 1313403 | LLQYGSFCTQLNRAL |
|  |  | 1313689 | YGSFCTQLNRALTGI |
|  |  | 1313732 | NLLLQYGSFCTQLNR |
|  |  | 1313796 | TQLNRALTGIAVEQD |
|  |  | 1313797 | TQLNRALTGIAVEQDKNTQ |
|  |  | 1313960 | TQLNRALTGIAVEQDKNTQEVFA |
|  |  | 1313987 | RALTGIAVEQDKNTQ |
|  |  | 1314023 | KNTQEVFAQVKQIYK |
|  |  | 1325172 | TQEVFAQVKQIYKTPPIK |
|  |  | 1329362 | VFAQVKQIYKTPPIK |
|  |  | 1329414 | VKQIYKTPPIKDFGGFNF |
|  |  | 1329491 | KQIYKTPPIKDFGGF |
|  |  | 1329496 | DFGGFNFSQILPDPS |
|  |  | 1329558 | FGGFNFSQILPDPSK |
|  |  | 1329628 | FGGFNFSQILPDPSKPSKR |
|  |  | 1329820 | GFNFSQILPDPSKPSKRS |
|  |  | 1329862 | NFSQILPDPSKPSKR |
|  |  | 1329957 | NFSQILPDPSKPSKRSFIE |
|  |  | 1330028 | NFSQILPDPSKPS |
|  |  | 1330030 | ILPDPSKPSKRSF |
|  |  | 1330178 | LPDPSKPSKRSFIEDLLF |
|  |  | 1330243 | KPSKRSFIEDLLFNK |
|  |  | 1330284 | KPSKRSFIEDLLFNKV |
|  |  | 1330296 | KPSKRSFIEDLLFNKVTLADA |
|  |  | 1330305 | SKRSFIEDLLFNKVTLADA |
|  |  | 1330361 | SKRSFIEDLLFNKVTLAD |
|  |  | 1330379 | RSFIEDLLFNKVT |
|  |  | 1332785 | RSFIEDLLFNKVTLA |
|  |  | 1383744 | SFIEDLLFNKVTLAD |
|  |  | 1388273 | SFIEDLLFNKVTLADA |
|  |  | 1392147 | IEDLLFNKVTLADAG |
|  |  | 1410559 | DLLFNKVTLADAGFIKQY |
|  |  | 1432856 | LLFNKVTLADAGFIK |
|  |  | 1435610 | VTLADAGFIKQYGDC |
|  |  | 1436695 | AGFIKQYGDCLGDIA |
|  |  | 1437846 | FIKQYGDCLGDIA |
|  |  | 1448898 | LGDIAARDLICAQKF |
|  |  | 1462784 | ARDLICAQKFNGLTV |
|  |  | 1465145 | FNGLTVLPPLLTDEM |
|  |  | 1471755 | LTDEMIAQYTSALLA |
|  |  | 1479937 | TDEMIAQYTSALLAG |
|  |  | 1482748 | MIAQYTSALLAGTIT |
|  |  | 1484768 | AQYTSALLAGTITSG |
|  |  | 1491922 | TITSGWTFGAGAALQ |
|  |  | 1498219 | GWTFGAGAALQIPFA |
|  |  | 1501129 | GWTFGAGAALQIP |
|  |  | 1505853 | WTFGAGAALQIPFAM |
|  |  | 1508891 | GAGAALQIPFAMQ |
|  |  | 1518044 | GAALQIPFAMQMAYR |
|  |  | 1518045 | ALQIPFAMQMAYRFNGIGV |
|  |  | 1518321 | QIPFAMQMAYRFNGI |
|  |  | 1518322 | IPFAMQMAYRFNGIGVTQNV |
|  |  | 1532692 | IPFAMQMAYRFNGIG |
|  |  | 1537200 | PFAMQMAYRFNGIGV |
|  |  | 1598225 | QMAYRFNGIGVTQNV |
|  |  | 1599614 | VTQNVLYENQKLIAN |
|  |  | 1599891 | QNVLYENQKLIANQF |
|  |  | 1600818 | QNVLYENQKLIAN |
|  |  | 1601336 | LYENQKLIANQFNSA |
|  |  | 1601746 | YENQKLIANQFNSAI |
|  |  | 1602664 | YENQKLIANQFNSAIGKIQ |
|  |  | 1603889 | KLIANQFNSAIGKIQ |
|  |  | 1604361 | KLIANQFNSAIGK |
|  |  | 1604374 | IGKIQDSLSSTASAL |
|  |  | 1604500 | GKIQDSLSSTASALGKLQ |
|  |  | 1605081 | DSLSSTASALGKLQD |
|  |  | 1605379 | GKLQDVVNQNAQALNT |
|  |  | 1605510 | GKLQDVVNQNAQALNTLVKQL |
|  |  | 1607628 | VVNQNAQALNTLVKQL |
|  |  | 1608726 | NQNAQALNTLVKQLSSNF |
|  |  | 1613044 | NQNAQALNTLVKQLSSNFG |
|  |  | 1614938 | AQALNTLVKQLSSNF |
|  |  | 1615090 | TLVKQLSSNFGAISS |
|  |  | 1615570 | TLVKQLSSNFGAI |
|  |  | 1615654 | QLSSNFGAISSVLND |
|  |  | 1615800 | ISSVLNDILSRLD |
|  |  | 1616213 | VLNDILSRLDKVEAE |
|  |  | 1616645 | KVEAEVQIDRLITGRL |
|  |  | 1617118 | KVEAEVQIDRLITGRLQSLQT |
|  |  | 1617939 | EAEVQIDRLITGRLQSLQ |
|  |  | 1618992 | VQIDRLITGRLQSLQ |
|  |  | 1619782 | VQIDRLITGRLQSLQT |
|  |  | 1619999 | RLITGRLQSLQTYVTQQL |
|  |  | 1621419 | LITGRLQSLQTYVTQ |
|  |  | 1621623 | LQSLQTYVTQQLIRA |
|  |  | 1621710 | QSLQTYVTQQLIRAAEIR |
|  |  | 1621919 | LQTYVTQQLIRAAEI |
|  |  | 1622602 | TYVTQQLIRAAEIRA |
|  |  | 1623119 | TQQLIRAAEIRASANLAA |
|  |  | 1624375 | QQLIRAAEIRASANL |
|  |  | 1624434 | QLIRAAEIRASANLAATK |
|  |  | 1624638 | QLIRAAEIRASANLA |
|  |  | 1625152 | AEIRASANLAATKMS |
|  |  | 1661541 | AEIRASANLAATKMSECV |
|  |  | 1672502 | EIRASANLAATKM |
|  |  | 1706856 | SANLAATKMSECVLG |
|  |  | 1870815 | ATKMSECVLGQSKRV |
|  |  | 1870822 | ECVLGQSKRVDFCGK |
|  |  | 1871461 | DFCGKGYHLMSFPQS |
|  |  | 1871542 | APHGVVFLHVTYVPA |
|  |  | 1871762 | VFLHVTYVPAQEKNF |
|  |  | 1871830 | TYVPAQEKNFTTAPA |
|  |  | 1871836 | QEKNFTTAPAICHDG |
|  |  | 1871880 | EKNFTTAPAICHDGKAHF |
|  |  | 1872160 | TTAPAICHDGKAHFP |
|  |  | 1872219 | TAPAICHDGKAHFPR |
|  |  | 1872258 | PAICHDGKAHFPREGVFV |
|  |  | 1872338 | KAHFPREGVFVSNGTHWFVT |
|  |  | 1872350 | THWFVTQRNFYEPQIITT |
|  |  | 1872421 | HWFVTQRNFYEPQII |
|  |  | 1872422 | HWFVTQRNFYEPQ |
|  |  | 1872550 | HWFVTQRNFYEPQIITTDN |
|  |  | 1872649 | QRNFYEPQIITTDNT |
|  |  | 1872669 | RNFYEPQIITTDNTFVSG |
|  |  | 1873053 | FYEPQIITTDNTFVSGNCD |
|  |  | 1873074 | EPQIITTDNTFVSGN |
|  |  | 1873075 | QIITTDNTFVSGN |
|  |  | 1873095 | TTDNTFVSGNCDVVI |
|  |  | 1873164 | TDNTFVSGNCDVVIG |
|  |  | 1873165 | FVSGNCDVVIGIV |
|  |  | 1873239 | GIVNNTVYDPLQPEL |
|  |  | 1873332 | TVYDPLQPELDSFKE |
|  |  | 1873602 | LQPELDSFKEELDKY |
|  |  | 1873624 | KEELDKYFKNHTSPD |
|  |  | 1873679 | SPDVDLGDISGINAS |
|  |  | 2144790 | GINASVVNIQKEIDR |
|  |  | 2145027 | VVNIQKEIDRLNEVA |
|  |  | 2145131 | KEIDRLNEVAKNLNE |
|  |  | 2145187 | KYEQYIKWPWYIWLG |
|  |  | 2150484 | YEQYIKWPWYIWLGF |
|  |  | 2150598 | CMTSCCSCLKGCCSC |
|  |  | 2150606 | DDSEPVLKGVKLHYT |
|  |  |  |  |
