# Supplementary figures and images for: Immune targets to stop future SARS-CoV-2 variants
Source: Microbiol Spectr. 2023 Nov 15;11(6):e02892-23. doi: 10.1128/spectrum.02892-23 (PMC10714790; doi:10.1128/spectrum.02892-23)

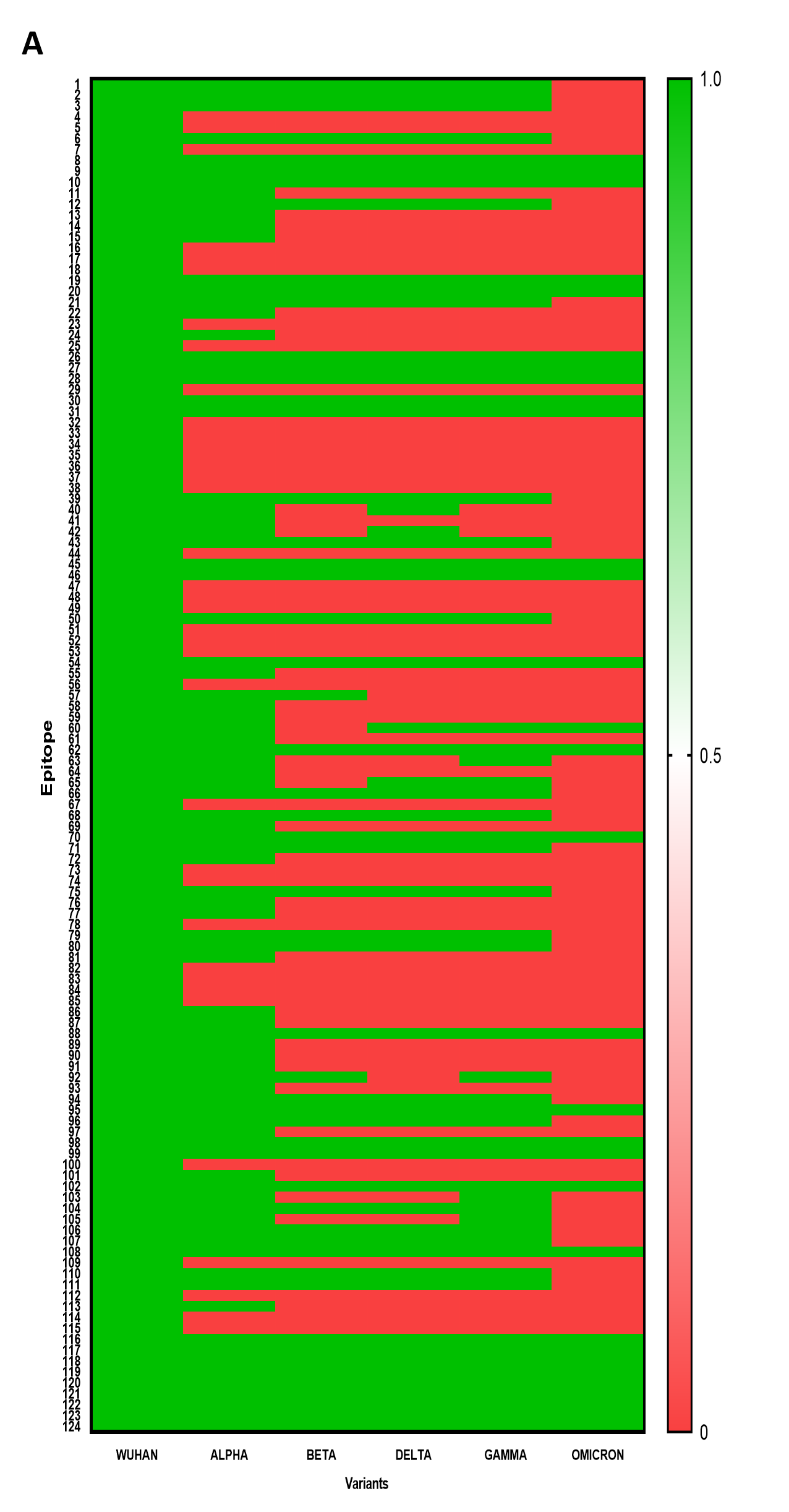

Supplement: Fig. S1 — Heat map of NAb epitopes fully conserved in Wuhan (WT), Alpha, Beta, Gamma, Delta, and Omicron VOCs. [file spectrum.02892-23-s0004.tif]
